# Geological sketch map and implications for ice flow of Thwaites Glacier, West Antarctica, from integrated aerogeophysical observations

**DOI:** 10.1126/sciadv.adf2639

**Published:** 2023-05-31

**Authors:** Tom A. Jordan, Sarah Thompson, Bernd Kulessa, Fausto Ferraccioli

**Affiliations:** ^1^ British Antarctic Survey, Cambridge, UK.; ^2^ Australian Antarctic Program Partnership, Institute for Marine and Antarctic Studies, University of Tasmania, Hobart, Tasmania, Australia.; ^3^ School of Biosciences, Geography and Physics, Swansea University, Swansea, UK.; ^4^ School of Geography, Planning, and Spatial Sciences, University of Tasmania, Hobart, Australia.; ^5^ Istituto Nazionale di Oceanografia e di Geofisica Sperimentale, Borgo Grotta Gigante, Sgonico, Italy.

## Abstract

The geology beneath Thwaites Glacier, the Antarctic glacial catchment most vulnerable to climate change, is unknown. Thwaites Glacier lies within the West Antarctic Rift System, but details of the subglacial geology relevant to glacial flow, including sediment availability, underlying lithology, and heat flux, are lacking. We present the first sketch map of the subglacial geology of Thwaites Glacier, interpreted from maps of airborne gravity, magnetic and radar data, supported by 2D models and 3D inversion of subsurface properties, and the regional geological context. A zone of Cretaceous mafic magmatism extending ~200 km inland from the coast is interpreted, while sedimentary basins are restricted to a region 150 to 200 km inboard of the coast, underlying just 20% of the catchment. Several granitic subglacial highlands are identified, forming long-lived topographic highs. Our geological interpretation places constraints on the basal properties of Thwaites Glacier, laying the foundation for both improved predictions of ice sheet change and studies of West Antarctic tectonics.

## Teaser

Geological sketch map provides insight into the basal conditions controlling the flow of the massive Thwaites Glacier.

## INTRODUCTION

### Importance of Thwaites Glacier and underlying geology for ice flow

**Fig. 1.**
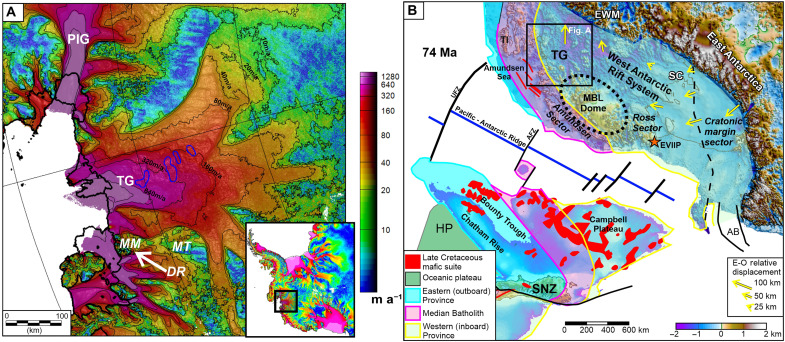
Glaciological and tectonic setting of study area. (**A**) Ice velocity of Thwaites Glacier (TG) (*69*). Ice shelf front and grounding lines are marked by thick black lines (Antarctic Digital Database 2021), and velocity contours are marked by thin black lines. Dynamic subglacial lakes outlined in blue (*70*). Mt. Murphy (MM) and Mt. Takahe (MT) are prominent Quaternary shield volcanoes. Dorrel Rock (DR) is an outcropping 35-Ma gabbro (*45*). PIG, Pine Island Glacier. Inset shows Antarctic location (black box) and ice velocity. (**B**) Tectonic setting of Thwaites Glacier in the context of West Antarctic provinces (*12*) with geology of the New Zealand conjugate margin reconstructed to its ~74-Ma post-breakup position (*16*), including South Island New Zealand (SNZ) and the Hikurangi Plateau (HP). Red lines in the Amundsen Sea mark magnetic anomalies attributed to mafic magmatism during continental breakup (*20*). Yellow arrows show Eocene to Oligocene (E-O) West/East Antarctic relative displacement (*22*). Dotted line outlines MBL dome. Dashed line separates sectors within the West Antarctic Rift System. Orange star marks outcrops of Anorogenic granites on Edward VII Peninsula (EVIIP) (*21*). EWM, Ellsworth Whitmore Mountains; SC, Siple Coast; TI, Thurston Island; UFZ, Udintsev Fracture Zone; AFZ, Antipodes Fracture Zone; AB, Adare Basin.

The West Antarctic Ice Sheet (WAIS) has been losing mass at an accelerating rate and is feared to be on a similar trajectory of rapid retreat as it was in previous warm periods (*1*). Understanding of the processes and time scales of WAIS mass loss is limited. However, Thwaites Glacier (Fig. 1A) has seen increasing ice velocity, grounding line retreat, and a doubling of its contribution to sea level rise since the 1990s, indicating that it is one of the most important and sensitive WAIS catchments (*1*). Thwaites Glacier is prone to marine ice sheet instability as it has a retrograde bed slope extending ~450 km to the WAIS’s ice divide, while its ~150-km-long grounding line and minimal buttressing ice shelf leave it vulnerable to changes in ocean circulation (*1*). Thwaites Glacier’s present-day grounding line is perched and stabilized on a transverse bedrock sill (*2*), although irreversible retreat from this sill may already be underway (*3*). Extensive retreat of Thwaites Glacier would affect the entire WAIS, incurring global average sea level rise of several meters in the coming decades to centuries (*1*).

Thwaites Glacier differs from other WAIS ice streams in that ice generally flows across, rather than along, the tectonic fabric (*2*). Linkages between underlying geology and ice dynamics may therefore differ from other WAIS catchments in their influence on the glacier’s current form and future stability. The onset and maintenance of WAIS ice-stream flow is typically associated with sedimentary basins (*4*, *5*), as rheologically weak marine muds or soft glacial tills derived from such basins can accommodate fast ice flow by deformation, in contrast to “sticky” bedrock sills, which may retard ice flow. Predicting the spatial patterns and time scale of Thwaites Glacier retreat and ensuing sea level rise therefore depends on knowing the sediment distribution and associated basal roughness, which are controlled by the underlying geological template.

The impacts of the underlying geology on Thwaites Glacier flow may be compounded by subglacial volcanism (*6*–*8*) and associated deeper lithospheric processes, increasing basal geothermal heat flux (*9*) and favoring enhanced basal melting. Basal melting, possibly supplemented by groundwater stored in underlying sedimentary basins (*10*, *11*), can promote subglacial sediment erosion, transport, and soft till deposition, all factors associated with enhanced ice flow. The region’s tectonic and magmatic evolution therefore has direct and important consequences for future rates of ice loss and sea level rise from Thwaites Glacier.

### Geological and tectonic setting

Thwaites Glacier lies within the West Antarctic Rift System/Marie Byrd Land (MBL) province of West Antarctica (Fig. 1B). Before rifting, this province, along with parts of Zealandia, was an ocean-continent convergent margin from ~500 to ~105 million years (Ma) (*12*, *13*). The inboard part of the province beneath Thwaites Glacier is part of the Ross Sector, composed of metamorphosed continental margin turbidites of the Swanson Formation deposited around 505 Ma, intruded by ~375- to ~250-Ma granitoids (*14*). These rocks were deposited and intruded along the active margin of Gondwana, which extended at least from the Lachlan Fold Belt of Australia to Mt. Murphy on the flank of Thwaites Glacier in Antarctica. Extensive deep-seated subduction-related magmas emplaced 250 to 105 Ma form the New Zealand Median Batholith, which continues into the outboard Amundsen Sector of West Antarctica (Fig. 1B) (*13*–*16*), and may continue into the Thurston Island region (*17*). Slowing and ultimate cessation of subduction due to the collision of the oceanic Hikurangi Plateau outboard of Zealandia 110 to 90 Ma triggered ~600 km of continental extension in West Antarctica (*12*). Extension associated with this initial phase of continental rifting exhumed mid-crustal metamorphic core complexes across the inboard Ross Sector (*18*). A subsequent phase of continental rifting began ~94 Ma and culminated with breakup and oceanic spreading between Zealandia (Campbell Plateau/Chatham Rise) and West Antarctica 89 to 85 Ma (Fig. 1B) (*19*).

During the later stage of continental rifting, as the system moved toward continental breakup, it is suggested that a series of mafic magmatic bodies were emplaced along the Amundsen Sea and Zealandia conjugate margins, as revealed by linear magnetic anomalies (*16*, *20*). Magnetic anomalies with a similar trend are noted beneath parts of the Thwaites Glacier catchment (*7*). Magnetic susceptibility measurements across New Zealand show that basaltic-gabbroic rocks typically have susceptibilities one to two orders of magnitude greater than most of the sampled rhyolitic-granitic igneous rocks, supporting a mafic—rather than felsic—interpretation for the magnetic sources (*16*). Other exposed syn-rift igneous rocks include 95- to 100-Ma anorogenic granites on the Edward VII Peninsula (Fig. 1B) (*21*). In contrast with the mafic rift-related rocks, the anorogenic granites have low measured magnetic susceptibility (*21*).

After the main Cretaceous rifting events, West Antarctica underwent further continental extension related to oceanic spreading within the adjacent Adare Basin 43 to 26 Ma (Fig. 1B) (*22*). The pole of rotation close to the Central Transantarctic Mountains implies that extension in the Adare Basin was accompanied by ~90-km oblique convergence in the Thwaites Glacier region (Fig. 1B) (*22*). Subsequent oblique movement of ~36 km from 26 to ~10 Ma (*22*) is potentially linked with later phases of localized West Antarctic extension (*23*). Volcanism attributed to upwelling hot asthenospheric mantle continued into the Holocene (<10,000 years), forming large shield volcanoes across the MBL Dome (*24*) and several recently active subglacial volcanoes (*7*).

### Existing geophysical characterization of the Thwaites Glacier catchment

The upper mantle of the West Antarctic Rift System, between 100- and 400-km depth, shows regional-scale heterogeneity in seismic velocity based on modeling relative travel times from teleseismic earthquakes (*25*). Low *P*- and *S*-wave velocities, relative to the model mean, beneath the MBL Dome are interpreted to reflect upwelling of warm asthenospheric mantle, while further inboard faster velocities suggest cooler mantle (*25*). Alternative Rayleigh wave tomography methods confirm this pattern, with higher *P*- and *S*-wave velocities beneath the interior of Thwaites Glacier linked to cooler lithospheric mantle or depletion of the upper mantle by melting during rifting (*26*). Moho depths of 20 to 29 km, estimated from both seismic and gravity observations, confirm that the West Antarctic Rift System is highly extended, with a thickness about half that of the unextended East Antarctic continent (*23*, *27*, *28*).

Reconnaissance airborne gravity data revealed linear free-air anomalies uncorrelated with specific topographic features, but broadly parallel to the topographic sill at the mouth of Thwaites Glacier (*29*). It was suggested that the underlying geology caused across-flow bands in ice sheet basal shear stress (*29*), clearly seen by later studies (*30*). High-resolution swath radar beneath the trunk of Thwaites Glacier (*31*) shows elongate flow parallel bedforms due to soft sediment and contrasting areas of rugged topography with fewer shorter lineations. Coincident seismic reflection surveys (*32*, *33*) indicate soft and hard material, and subglacial water at the ice-bed interface, with harder bed generally correlating to the rugged topography. The complex pattern of bed conditions revealed by these localized studies likely enhances and retards the ice flow in different areas. We explore the likely controlling role played by the underlying geology.

Elevated geothermal heat flux values of between 70 and 130 mWm^−2^ relative to typical continental crust (65 mWm^−2^) (*34*) have been suggested for the Thwaites Glacier region. These results come from a range of different techniques, including spectral analysis of magnetic anomalies (*9*), analysis of radar reflectivity and water routing (*35*), and analysis of seismic data (*36*). A regional heat flux of ~97 mW m^−2^ has been inferred from the thin crust indicated by gravity data, with higher values predicted in MBL, but major uncertainty arising from the poorly constrained ages of key tectonic events was acknowledged (*27*). Although many estimates of heat flux have been made, inconsistent spatial patterns are observed, contested assumptions are made for each method, and direct in-rock measurements of heat flux to constrain models are lacking, making confident choice of specific heat flux values or models challenging.

It is evident that lack of knowledge of the geology beneath Thwaites Glacier both hampers our understanding of the regional tectonic structure and limits our ability to confidently predict how interactions between the cryosphere and the rocks beneath will influence future glacial behavior. To provide a holistic view of the Thwaites Glacier catchment, we have created compilations of airborne radar, magnetic, and gravity data, outlined in Materials and Methods. These compilations are analyzed and interpreted to provide a geological sketch map, together with an interpretation of the subglacial geology and discussion of the implications for ice flow.

## RESULTS

### Geophysical mapping

**Fig. 2.**
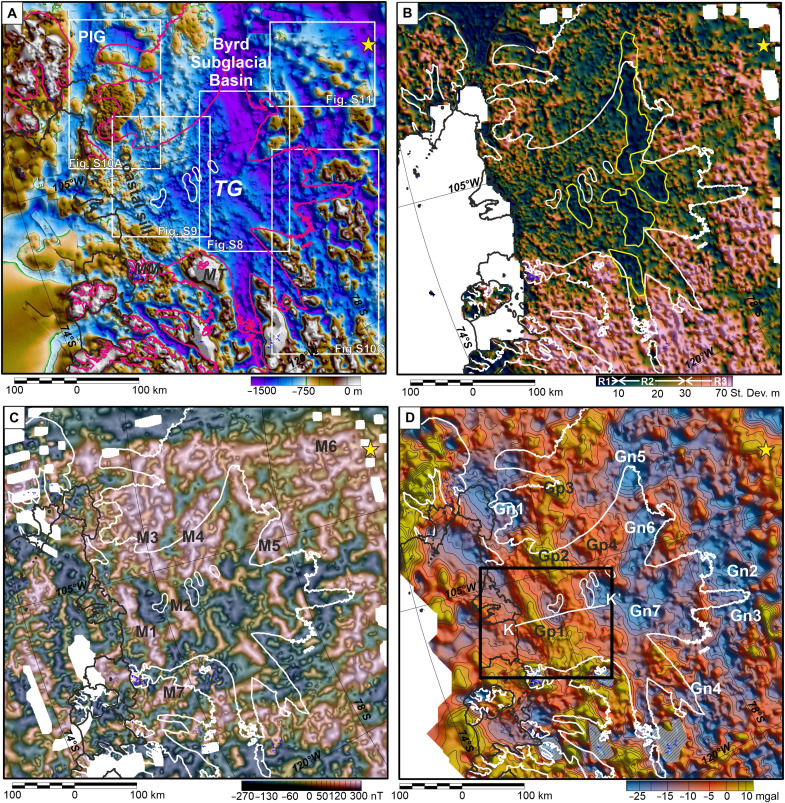
Main geophysical datasets. (**A**) Topographic compilation. White boxes locate supplementary figures. Pink line, 40 m a^−1^ ice velocity contour. Yellow star marks ~500-m-thick sediments from receiver functions (*39*). (**B**) Interpolated along-track bed roughness from airborne radar, with smooth areas outlined in yellow. The thresholds of low, intermediate, and high roughness domains (R1 to R3) are marked on color bar. (**C**) Reduced-to-the-pole magnetic anomaly map, continued to 500 m above the sea/ice surface. Anomaly edges from TDX enhancement are shaded black. Anomalies M1 to M7 are type examples discussed in the text. (**D**) Residual Airy isostatic gravity anomalies with 5-mgal contour interval. White line locates model K-K′ (Fig. 4). Black box locates 3D inversion (Fig. 5). Type examples include Gp1 to Gp3 positive gravity anomalies, Gn1 to Gn4 negative gravity anomalies associated with elevated topography, and gravity anomalies Gn5 to Gn7 that occur in low-lying regions, often associated with smooth bed. Hashed gray regions in grid south mark artifacts due to the lack of gravity data coverage over high topography.

Topographic and roughness maps derived from airborne radar data show three broad physiographic domains (Fig. 2, A and B). The domain with the highest roughness, >30-m along-track standard deviation in bed elevation, corresponds to the inboard incised highlands. The intermediate roughness domain (10 to 30 m standard deviation) extends from the inboard highlands to the coast. The final domain shows extremely low roughness, with a standard deviation of <10 m. Within the Thwaites Glacier catchment, such low roughness areas are confined to elongate bands 150 to 200 km inboard from the coast.

Magnetic anomalies show a complex pattern, with distinct trends in different areas (Fig. 2C and fig. S4). Within the study area, we identify a series of “type” magnetic anomalies (M1 to M7), which are extensive (>50 km long), have high amplitude (>300 nT), show distinct trends, and, in some cases, correlate with other geophysical signatures. The edges of the anomalies are indicated by peaks in the horizontal tilt angle (TDX) digital enhancement, shown as dark shading (Fig. 2B). The type magnetic anomalies are described here, and their sources are subsequently discussed. Other magnetic anomalies that match the character and geophysical correlations of the type anomalies are interpreted to have the same geological source. In the downstream area of Thwaites Glacier, two anomalies (M1 and M2) lie at high angle to the ice flow. These anomalies have a grid north-northwest trend and may link with anomalies M3 and M4, which have a generally north-northeast trend and form part of a more highly magnetic region with a broadly grid north to south trend (fig. S4). Further inboard, magnetic anomalies M5 and M6 lie within the rough incised highlands and show generally grid east to west, to east-northeast to west-southwest trends, similar to anomaly M7 close to Mt. Murphy and Mt. Takahe (fig. S4).

The study area includes a complex pattern of residual Airy isostatic gravity anomalies with a range of signs and amplitudes (Fig. 2D). The key type gravity anomalies discussed in this study were selected on the basis of their amplitude (generally >±20 mgal) and correlation with topographic or other geophysical signatures. The boundaries of these anomalies are loosely constrained on the basis of the amplitude and gradient of the gravity field. The type gravity anomalies include, first, positive gravity anomalies Gp1 to Gp4, which have peak amplitudes of 0 to 10 mgal, ~20 mgal above the surrounding mean level, and are spatially coincident with magnetic anomalies M1 to M4. Second, negative isostatic anomalies Gn1 to Gn4 with amplitudes of −20 to −35 mgal are associated with topographic highlands. Last, negative anomalies Gn5 to Gn7, with an average amplitude of −30 mgal, are associated with low-lying topography and often with smooth bed. We note that many additional gravity anomalies are present with equivalent amplitude to the type anomalies; however, a lack of precise correlation with other signatures means that it is not currently possible to interpret their origins.

### Depth-to-source estimates

**Fig. 3.**
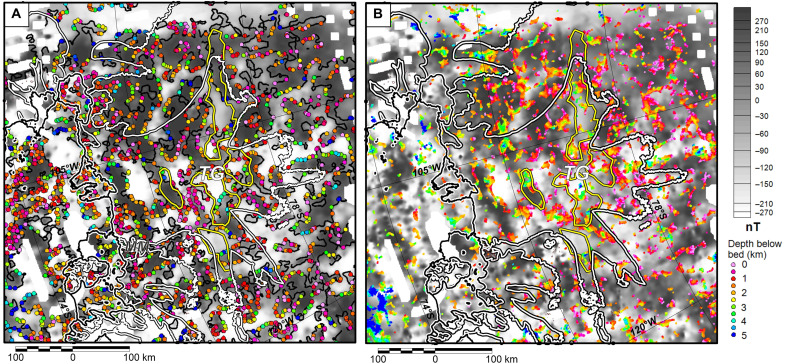
Magnetic source locations beneath the ice sheet bed from 3D depth estimates, overlain on reduced-to-pole aeromagnetic anomaly data. Yellow lines show edges of sedimentary basins independently interpreted from bed roughness (Fig. 2B). White lines mark 40 m a^−1^ ice velocity contour, defining approximate onset of enhanced flow within the Thwaites Glacier catchment. (**A**) Tilt depth solutions along 0° tilt contour (black line) marking theoretical source margins. (**B**) 3D extended Euler solutions. Both techniques indicate that most sources are close to the ice-bed interface.

Estimates of the depth of magnetic sources based on three-dimensional (3D) methods suggest that sources are typically between ~0 and 5 km below the ice-bed interface, with isolated estimates at 8 km or below (Fig. 3). Tilt depth solutions (Fig. 3A), which are tied to the margins of the main anomalies, show deeper solutions compared to the 3D Extended Euler method (Fig. 3B), which returns many more solutions. However, along the anomaly margins, there is a broad correspondence in the results, with a standard deviation of 790 m between the two methods. These depth-to-source estimates show little evidence of a thick and extensive post-tectonic sedimentary basin exclusively filled with nonmagnetic sediment overlying the magnetic sources across the Thwaites Glacier catchment. Rather, they suggest that the magnetic sources are generally close to the ice-bed interface, as noted by previous West Antarctic studies (*6*). This could be indicative of thin sediments or extensive volcanic rocks or intrusions within any sedimentary sequences.

**Fig. 4.**
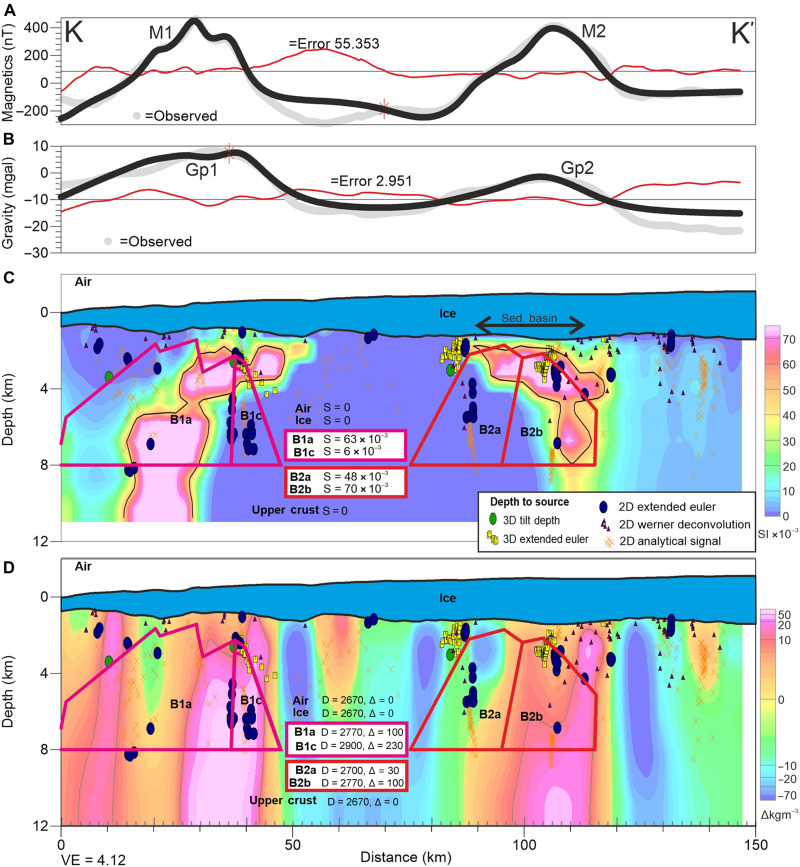
Profile K-K′ showing magnetic and gravity anomalies together with 2D forward model, depth-to-source results, and sections from 3D inversions for susceptibility and density over key anomalies. (**A**) Observed and forward calculated magnetic anomalies M1 and M2. (**B**) Observed and forward calculated residual Airy isostatic gravity anomalies Gp1 and Gp2. (**C**) 2D forward model of crustal structure. Body properties are magnetic susceptibility (S) in SI units. Symbols denote 2D and 3D magnetic depth-to-source estimates used to guide the forward model. Background colors show results of 3D inversion for apparent magnetic susceptibility, constrained to be >0 and <75 × 10^−3^ SI. (**D**) 2D forward model overlain with results of 3D inversion for rock density contrast, constrained to lie between ±1000 kg m^−3^. Forward model body properties (D) are density in kg m^−3^, with density contrast (Δ) against a background of 2670 kg m^−3^ also shown.

Depth-to-magnetic source results from profile data show a broad scatter of solutions (Fig. 4 and fig. S6). However, when superimposed, clusters of 2D solutions become apparent, suggesting magnetic sources between the ice bed and ~1 km depth. Additional depth estimates placing sources above the bed are disregarded, but are indicative of the generally shallow results returned by all techniques. The 2D results are consistent with 3D depth estimates within 5 km of the profiles (Fig. 4).

### 2D modeling and 3D inversion

The 2D forward models of upper crustal structure fit the observed Airy isostatic gravity and magnetic anomalies with source bodies ~6 km thick, with their tops located close to the ice bed, broadly consistent with depth-to-source solutions (Fig. 4). Bodies with a simple outward-dipping geometry provided an adequate fit to the data, and more complex bodies are hard to justify given the lack of independent constraints. The modeled source bodies generally had both high susceptibility and density, although variation of density and susceptibility within the bodies was required to fit to the anomalies, indicating the internal complexity of the sources.

**Fig. 5.**
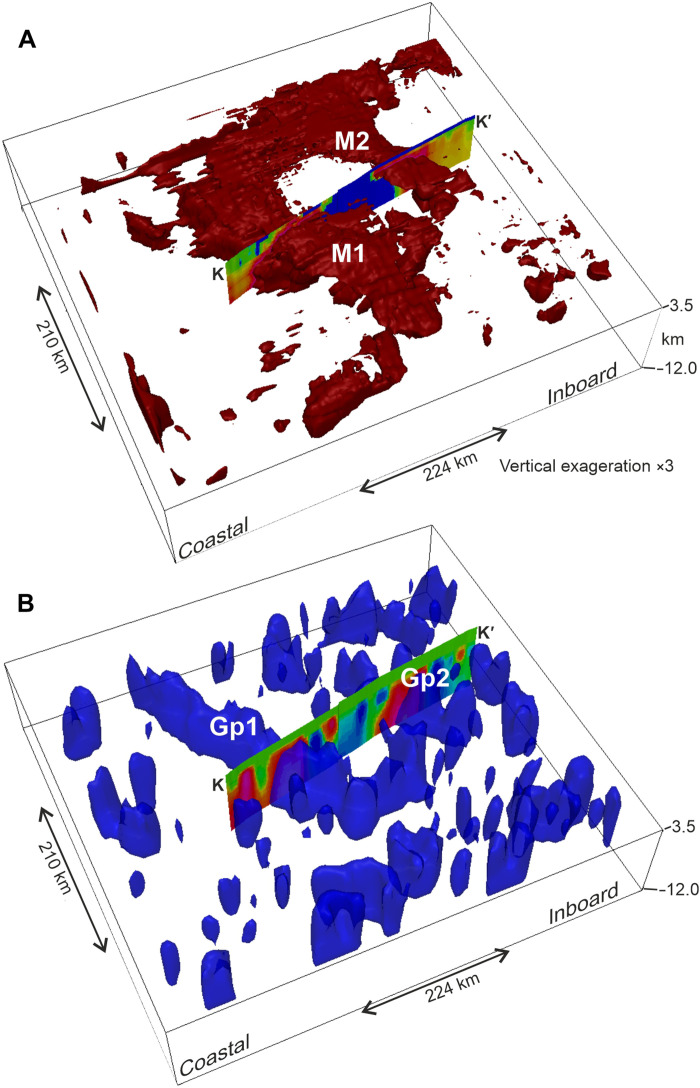
Oblique view of inversion results constrained by magnetic or Airy isostatic gravity anomalies. Inversion area located in Fig. 2D. Sections show susceptibility or density profiles extracted from inversion output and shown in Fig. 4. (**A**) Magnetic model volume where apparent magnetic susceptibility is >50 × 10^−3^ SI. Main bodies are interpreted to reflect mafic intrusions giving rise to magnetic anomalies M1 and M2. (**B**) Density model volume where density >20 kg m^−3^ above background, giving rise to Airy isostatic gravity anomalies Gp1 and Gp2.

The results of the 3D inversion show relatively good agreement between the input and predicted magnetic and gravity fields (fig. S7). However, the amplitude of the negative magnetic anomaly between anomalies M1 and M2 is not matched as well as other parts of the model. This suggests that the inversion may require negative susceptibility in this region, indicative of a component of unmodeled magnetic remanence in this area. Estimates of magnetic source geometry and properties from 3D inversion generally predict high-susceptibility bodies lying within the area of the bodies used in the forward models, and broadly coincident with the locations of depth-to-source clusters (Figs. 4C and 5A). Independent 3D inversion of density structure from Airy isostatic gravity anomalies places dense bodies in similar regions, although the bodies derived from gravity data are less well resolved (Figs. 4D and 5B). As the 3D inversions have limited constraint, the absolute susceptibility or density values and precise source geometry is not considered robust. However, the correspondence of high-susceptibility bodies from 3D inversion with our 2D models suggests that the magnetic inversion results give a reasonable first approximation of the 3D structure (Fig. 5A). Assuming a threshold value of between 40 × 10^−3^ and 60 × 10^−3^ in the International System of Units (SI) suggests that within the inversion domain, 10 to 14% of the crust shallower than 10 km can be attributed to the high magnetic susceptibility sources. Given the nonunique nature of inversion results, this volume must be considered with caution. However, our maps show that magnetic sources underlie approximately 20% of the inversion region by area, and forward models suggest that bodies are ~6 km thick, giving an alternative but consistent estimate of ~12% of the upper 10 km of crust being formed from high-susceptibility material.

### Interpretation

**Fig. 6.**
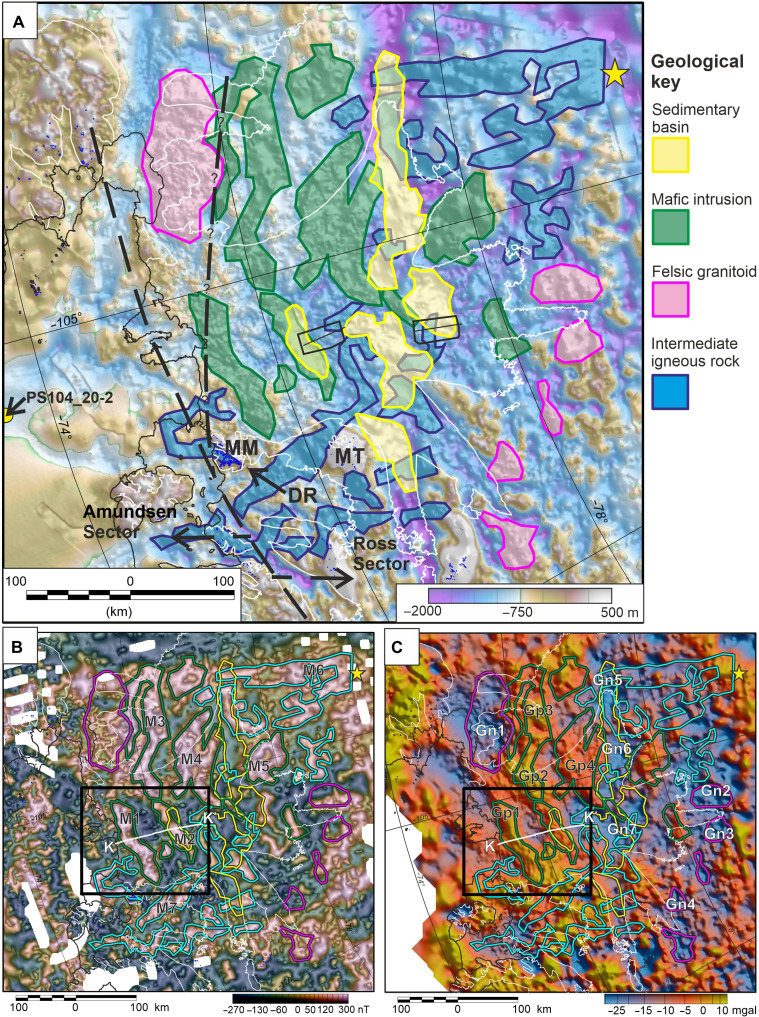
Interpreted geological sketch map. (**A**) Overlain on subglacial topography. Small black boxes and thin lines locate swath radar surveys and associated seismic profiles (*31*–*33*). PS104_20-2 locates marine drill site with Cretaceous sediments (*42*). Dashed line locates Ross/Amundsen Sector boundary (*14*) and our proposed alternative route (?). (**B**) Overlain on magnetic anomalies shaded with TDX edge enhancement. Black box and white profile locate 3D inversion and 2D model, respectively. (**C**) Overlain on residual Airy isostatic gravity anomalies.

We interpret four geological units based on our aerogeophysical maps and models (Fig. 6). Each unit has a specific combination of geophysical characteristics, outlined in Table 1 and fig. S2B. These geophysical characteristics are interpreted as reflecting distinct lithologies. A more speculative interpretation is made of the tectonic origin of each unit in the context of West Antarctic evolution, including information from sparse rock exposures, subglacially derived rocks, and knowledge of the conjugate margin in New Zealand. We note that our geological sketch map (Fig. 6) shows gaps between interpreted units. These regions do not show clear geophysical signatures, allowing us to confidently interpret the underlying geology. Our working assumption would be that these areas are composed of the metasedimentary basement of the Ross Sector of West Antarctica such as the Swanson Formation or higher metamorphic grade equivalents (*14*).

**Table 1.** Geophysical characteristics of the interpreted lithologies. The most important signatures for each interpreted lithology are underlined. “NC” indicates that a geophysical observation has no consistent correlation with the other geophysical signatures in the areas where a specific lithology is interpreted.

Observation lithology	Radar topography	Radar roughness	Airy isostatic gravity anomaly	Magnetic anomaly
Sedimentary basin	Low-lying basin	Very smooth	Negative*	NC
Mafic intrusion	NC	Intermediate to rough*	Positive	Positive
Felsic granitoid	Highland	Rough	Negative	NC
Intermediate	NC	NC	NC	Positive

*The most downstream sedimentary basin is interpreted to overlie a mafic intrusion, giving a complex superposition of signatures.

### Sedimentary basins

The first unit we interpret is a series of elongate but relatively restricted sedimentary basins between 80 and 200 km long and ~30 km wide (Fig. 6 and fig. S8). The primary feature of these regions is low topographic roughness (standard deviation <10 m) (Fig. 2B). The presence of such smooth subglacial bed is often attributed to a drape of interglacial marine sediments (*37*). However, in the marine drape scenario, all of the Thwaites Glacier catchment should have received an equivalent sediment blanket and, hence, show equivalent low roughness. Instead, we attribute these localized areas of smooth bed to a combination of weak and deformable till at the ice-bed interface, coupled with underlying basins filled with weak and easily eroded sediments.

Weak and deformable till in the areas of low basal roughness is revealed by swath radar showing elongate till ridges known as Mega Scale Glacial Lineations (MSGLs) (fig. S8C) (*31*). Seismic measurements confirm that the MSGLs are soft tills (*32*, *33*). The thickness of the till layer is unknown; however, the MSGLs are ~100 m high, providing a minimum estimate for till thickness over the sedimentary basins, assuming that the MSGLs are sedimentary features. Linear features indicative of soft sediment deformation on the order of 1 to 5 km long are present outside the proposed sedimentary basins, but these structures are often fragmentary or have a distinct crag and tail appearance (fig. S8C). These structures are therefore distinct from the 12- to 20-km-long MSGLs, which dominate the subglacial geomorphology in the areas between apparent bedrock highlands. The linear features on the highlands may reflect packages of subglacial sediment, but their fragmentary nature indicates that there is not a thick continuous till layer, although a thin veneer over the entire region cannot be ruled out. Seismic data confirm that soft till is present as a sporadic drape in some more rugged regions (*32*, *33*), but the detailed radar observations show that it fails to blanket and obscure the rugged topography.

Reflection seismic observations do not image the bedrock beneath the till in the regions of smooth bed (Fig. 6) (*32*, *33*). However, associated negative Airy isostatic gravity anomalies of −10 to −15 mgal (Gn5 to Gn7) are consistent with underlying basins filled with relatively low density sediment (fig. S8B). Given densities between 2400 and 2500 kg m^−3^ for compacted sediments (*38*) and a background density of 2670 kg m^−3^, a simple Bouguer slab calculation suggests that the sediments are on the order of 0.9 to 2.1 km thick. This is much greater than expected for a drape of deforming till. Magnetic depth-to-source solutions suggests that the sediments may in places be 3 to 5 km thick (Fig. 3), but there is broad uncertainty in this estimate. A single receiver function observation (*39*) on the margin of the study area (Fig. 6) confirms that sediments 500 ± 250 m thick are present in parts of the Thwaites Glacier catchment. However, although relatively smooth, the bed roughness at this seismic station did not meet our threshold of <10-m standard deviation for definition as a sedimentary basin, suggesting that the specific seismically imaged sedimentary package may be relatively restricted in space. We also note that one of the extremely smooth areas interpreted as a sedimentary basin overlies positive Airy isostatic gravity anomaly Gp2 (Fig. 6C), in contrast to the upstream basins. We suggest that in this specific case, superposition of signals from an underlying body is masking the negative gravity signature of the sedimentary basin.

**Fig. 7.**
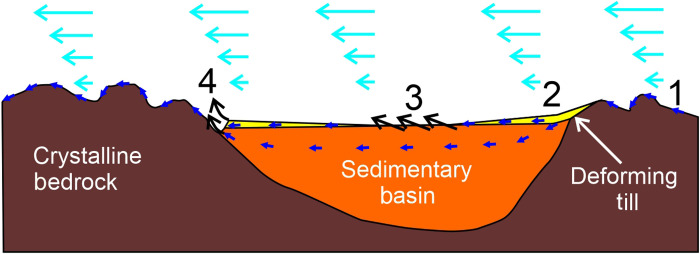
Sketch of till (yellow) erosion and transport processes across an underlying sedimentary basin (orange) bounded by metamorphic or igneous crystalline basement (brown). Ice flow, pale blue arrows; water flow, dark blue arrows; erosion, black arrows. Zone 1 is dominated by erosive scouring of crystalline bedrock by ice and subglacial water. Zone 2 is associated with deposition of soft lee-side till sediment tails (*31*), likely containing a high proportion of water (*2*). In zone 3 if/when underlying sedimentary rocks are exposed, upstanding topography is quickly removed by glacial erosion. Zone 4 includes an upstream moat cut into sediments abutting highlands formed of crystalline bedrock. In this setting, water may be squeezed out of sediments by local pressure gradients facilitating enhanced glacial erosion (*2*). Water in the resulting channelized system likely flows over and around the highlands cutting observed sinuous channels (fig. S7C).

A thick basin containing relatively poorly lithified sedimentary rocks underlying the areas of smooth bed provides a mechanism for how these regions gain and maintain their relatively smooth appearance (Fig. 7). Poorly lithified sedimentary rocks would not resist glacial erosion, so any locally developing highlands within a sedimentary basin would quickly erode, leaving a low-relief, low-friction surface. Eroded material would add to the deforming till layer, shielding the underlying sediments from further erosion. In contrast, surrounding regions with inferred crystalline (metamorphic or igneous) bedrock would resist erosion, leading to the development of the observed more rugged basal topography. Although somewhat speculative, this conceptual model helps explain why extremely smooth bed, the result of a modern glaciological process, is restricted to the areas of preexisting sedimentary basins.

The deforming till layer is likely an actively evolving feature associated with the current ice sheet (*2*). The age of any underlying sedimentary basins is harder to interpret. The absence of highlands in the smooth regions suggests that the underlying rocks are not well lithified. The metasedimentary rocks of the Swanson Formation or other metasedimentary rocks deposited and deformed within the Paleo Pacific convergent margin system before 110 Ma are therefore unlikely to form these basins. The highly deformed mid-Cretaceous and older basement across West Antarctica and New Zealand is truncated by an extensive low-relief erosion surface linked to uplift before rifting (*40*). The inferred poorly lithified sedimentary basins we identify likely postdate this erosion surface. In New Zealand, the post-erosion surface sediments include extensive nonmarine to shallow marine sediments generally younger than 103 Ma, with isolated sections up to 112 Ma old (*41*). Offshore from Thwaites Glacier, the oldest undeformed sedimentary rocks are Middle Cretaceous (121 to 83 Ma) recovered from drill site PS104_20-2 (Fig. 6A) (*42*). These rocks were deposited in an extensive continental rift system, dated in New Zealand to between 100 and 85 Ma (*40*, *41*). Seismic data show that the Middle Cretaceous sedimentary rocks offshore from Thwaites Glacier rest directly on the basement (*42*). Because the localized sedimentary basins we interpret are approximately parallel to the rifted continental margin, our preferred interpretation is that they reflect inboard relics of the broader Cretaceous rift–related sedimentary province identified both offshore from Thwaites Glacier and in New Zealand. An alternative model is that these basins formed in Eocene-Oligocene time, contemporary with the opening of the Adare Basin (Fig. 1B). However, the predicted Eocene-Oligocene extension direction is approximately parallel to the interpreted basins (Fig. 1B) (*22*), requiring a more complex transtensional origin. Irrespective of their age, the linear nature of these basins suggests that there is tectonic control on sediment preservation. We suggest that downfaulted basins allowed preservation of sediments, while equivalent material was eroded from the surrounding regions.

### Mafic intrusions

The second unit is interpreted as mafic intrusions (Fig. 6), marked by coincident positive Airy isostatic gravity and magnetic anomalies (Fig. 2 and fig. S9). Our 2D forward modeling (Fig. 4 and fig. S6) shows that the anomalies can be explained by geometrically simple dense upper crustal sources (30 to 230 kg m^−3^ denser than the background material) with relatively high magnetic susceptibility (48 × 10^−3^ to 70 × 10^−3^ SI). A mafic body would be a reasonable source for the modeled positive Airy isostatic gravity anomalies, given the required densities (*38*). Such a source lithology would also be consistent with the high magnetic susceptibility required by our forward models and inversions. Both intrusive and volcanic sources could be possible. However, we prefer an intrusive model as the thickness of the modeled bodies is large for a volcanic pile. Magnetic remanence in mafic rocks can generate localized strong positive and negative magnetic anomalies; however, in this case, the positive Airy isostatic gravity anomalies would not be expected to correlate just with the trace of the positive magnetic anomalies. In addition, although localized poor fit of the 3D inversion suggests that remanence may play some role (fig. S7), the overall amplitude of the observed magnetic anomalies fits well with the assumption of solely induced magnetism. Therefore, although remanence may play a part, it is reasonable to make the simplifying assumption during modeling that the observed magnetic anomalies are due predominantly to induced magnitization in high-susceptibility rocks, rather than remnant magnetization.

The age of the inferred mafic intrusions is unknown. Iceberg-rafted debris from Thwaites Glacier provide a range of candidate ages, but cannot identify specific intrusions and only reflect sources eroding at the ice-bed interface, so ages of subsurface bodies may be missing. Peaks in ^40^Ar/^39^Ar cooling-age abundance suggest that the dominant phases of tectonic activity were 100 Ma and 115 Ma, with a more minor peak at 35 Ma and a tail of ages stretching back over 300 Ma (*43*). As these ages are cooling ages, they may in part reflect uplift and cooling, or a metamorphic overprint, rather than magmatic emplacement ages. However, the abundance peaks do mark periods of more intense tectonic activity and, in other areas, have been shown to track peaks in zircon abundance, which are a more direct indicator of emplacement age (*44*). Although a few ages <25 Ma are noted, the overall suite of ages does not support an extensive syn-glacial Cainozoic subglacial volcanic suite beneath the Thwaites catchment, as suggested from magnetic data in other parts of the West Antarctic Rift System (*6*).

Given the abundance of ~100 Ma ages, the inferred mafic intrusions may relate to continental rifting at this time. Swarms of ~101 Ma mafic and intermediate dykes west of Thwaites Glacier support a model of magmatic continental breakup (*15*). A rift-related model is consistent with offshore magnetic anomalies interpreted as deep-seated mafic bodies associated with continental breakup (Fig. 1B) (*20*). The broad correspondence between offshore bodies and those we interpret as mafic intrusions was also noted by previous authors (*7*). Extensive, generally margin parallel, mafic bodies controlled by syn-rift structures related to Gondwana breakup are seen in the conjugate Chatham Rise and Campbell Plateau (Fig. 1B) (*16*). These conjugate structures, dated to ~84 Ma from exposures on remote islands, are slightly older than the oldest oceanic floor and offset from the final rifted margin, indicating that they reflect a failed rift structure (*16*). Together with our observations, these conjugate structures support the idea that mafic magmatism was widespread during the period between 100 and 84 Ma, preceding the breakup between West Antarctica and New Zealand. An alternative younger age of ~34 Ma for mafic magmatism is consistent with gabbros exposed at Dorel Rock (Fig. 6A) (*45*). Although a plausible source that cannot be ruled out, the correspondence in trend with offshore and conjugate magnetic anomalies, and the dominant detrital ages, leads us to prefer a Cretaceous age for the interpreted mafic intrusions.

### Felsic granitoids

The next unit is interpreted as a series of felsic granitoid intrusions. The geophysical signatures defining this unit are negative Airy isostatic anomalies associated with elevated topography (Gn1 to Gn4) (Fig. 6 and fig. S10). A range of sources including low-density sedimentary rocks and felsic (silica rich) igneous rocks can generate negative gravity anomalies. However, the association of negative gravity anomalies and elevated topography indicates a low-density but relatively hard to erode rock, more consistent with an igneous than a sedimentary source. These geophysical observations are similar to those in the Ellsworth Whitmore Mountains, where subglacial highlands including exposed granite peaks are associated with negative gravity anomalies (*46*).

The presence of granitic intrusions in the Thwaites Glacier catchment is consistent with outcrops on islands outboard of Pine Island Glacier, where granites are the dominant lithology (*15*). Ice-rafted debris from Pine Island and Thwaites Glaciers is also dominated by granitic clasts (*47*). The exposed granites are generally Late Cretaceous, reflecting culmination in arc magmatism before rifting (*15*, *48*). The subglacial granites we identify may therefore reflect a continuation of the New Zealand Median Batholith (Fig. 1B) (*16*). The poor correlation between the inferred granites and magnetic signatures (Fig. 6, B and C) is consistent with the observation that many of the conjugate Median Batholith granites in New Zealand have relatively low magnetic susceptibility (*16*). Although consistent with local rock exposure on offshore islands, the onshore continuation of the Median Batholith requires that the previously proposed boundary of the Amundsen and Ross sectors (*14*) lies further inboard beneath Thwaites Glacier (Fig. 6A). This interpretation is not possible for the smaller inboard felsic granitoids, which lie away from the proposed trace of the Median Batholith and well within the basement of the Ross Sector exposed at Mount Murphy (Fig. 6A). One possibility is that they are anorogenic granites similar to those emplaced on the Edward VII Peninsula (Fig. 1B). These rocks were emplaced during continental rifting ~95 to 100 Ma and also show low measured magnetic susceptibility (*21*). An alternative is that the interpreted inboard granites are part of an extensive Late Devonian to Late Carboniferous suite emplaced along the margin of Gondwana, including in the Lachlan Fold Belt of Australia, in Northern Victoria Land Antarctica and forming the New Zealand Karamea Suite (*49*). Further zircon dating and geochemical analysis of ice-rafted debris is required to constrain if granitic rocks associated with the Amundsen or Ross Sector dominate beneath the Thwaites and Pine Island Glaciers.

### Intermediate igneous rocks

The final unit is interpreted as an intermediate composition igneous rock, i.e., not strongly mafic or felsic in composition. This unit corresponds to magnetic anomalies >200 nT with no clear association with Airy isostatic gravity anomalies (Fig. 6 and fig. S11). In this situation, the magnetic source body must have a density similar to the surrounding rocks. The high magnetic susceptibility is more typical of an igneous rock. We cannot rule out extensive metamorphic reworking; however, an igneous protolith typically results in more magnetic metamorphic rocks than a sedimentary one. It is not clear whether the sources are volcanic or intrusive.

The age of the intermediate unit is unknown. The dominant grid east to west to northeast to southwest orientation contrasts with the trend of the proposed mafic intrusions (fig. S4), suggesting emplacement in a different tectonic setting. Leaky transfer faulting within a failed continental rift producing the mafic intrusions, as suggested on the conjugate margin (*16*), cannot be ruled out as a source for these rift orthogonal features. However, as high-density bodies are not indicated, we prefer an interpretation where the sources are distinct. One model is that the intermediate unit reflects more magnetic West Antarctic basement, predating the Cretaceous West Antarctic Rift System. Basement of this age would be consistent with the >115-Ma ages apparent in the detrital record (*43*). Alternatively, these anomalies may reflect subglacial intrusions associated with Cainozoic volcanism, as suggested in other parts of the West Antarctic Rift System (*6*). The inferred intermediate bodies lie approximately orthogonal to the Eocene-Oligocene and subsequent middle Neogene extension direction (*22*), suggesting that this late-stage event may be an important control. The spatial correlation of some of these anomalies with recent shield volcanoes, such as Mt. Takahe and Mt. Murphy (*24*), is consistent with a younger interpretation (Fig. 6). Both pre- and post-rift scenarios may be true across different parts of the study area.

## DISCUSSION

The geological sketch map we have produced is the first detailed picture of the subglacial geology of the Thwaites Glacier catchment. Our interpretation has wide-ranging implications for understanding how the region’s tectonic evolution affects the dynamics of the overlying ice sheet.

### Distribution of sedimentary rocks

The sedimentary basins inferred to contain thick preglacial sedimentary sequences within the Thwaites Glacier catchment, occur in a relatively narrow band 150 to 200 km inboard of the grounding line, forming ~20% of the bed in the catchment where ice is flowing faster than 40 m a^−1^. The location of these basins in the upper reaches of Thwaites Glacier is important as they can act as a source of deformable till capable of reducing basal friction and enhancing ice flow downstream (*4*). However, continuous sedimentary basins are likely a prerequisite for streaming flow (*5*). The discontinuous and spatially restricted nature of the sedimentary basins beneath Thwaites Glacier may help to explain the higher basal shear stress required to maintain its steeper surface slope compared to the Siple Coast, or Institute Ice Streams (*50*). These other catchments show lower surface slopes and are both underlain by sedimentary basins over 1000 m thick (*5*, *46*).

### Direct coupling of geological structures and glaciology

**Fig. 8.**
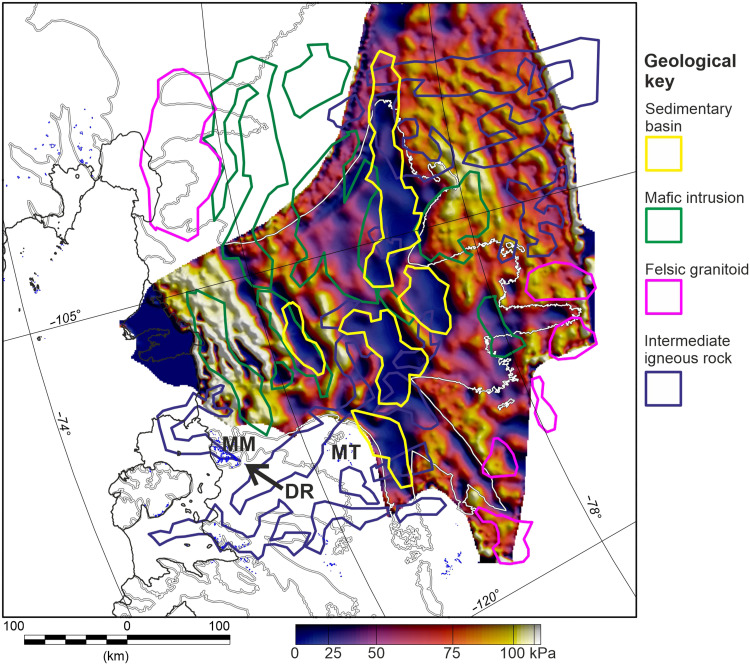
Modeled basal shear stress overlain with our geological sketch map. Basal shear stress is from a 3D thermomechanical ice sheet model with a simple flow relation for ice anisotropy, constrained by observed ice surface velocity (*51*). In upstream areas, low shear stress regions show close correlation with interpreted sedimentary basins, while in the downstream areas, the trend of the shear stress variation matches the trend of the interpreted subglacial geology outside the sedimentary basins.

We propose that Cretaceous rifting between West Antarctica and New Zealand dominates the subglacial geology beneath Thwaites Glacier (Fig. 6). The interpreted Cretaceous rift structures, including mafic intrusions and sedimentary basins, are likely associated with rift parallel fault systems running approximately orthogonal to current ice flow. Thwaites Glacier is characterized by bands of high and low basal shear stress 5 to 10 km wide (*30*, *51*), which parallel the underlying geological structures (Fig. 8). In the upper reaches of Thwaites Glacier, shear stress is low where we interpret sedimentary basins and higher in between. Further downstream, the bands of shear stress appear independent of the interpreted sedimentary basins, and do not show a simple correlation with subglacial topography, suggesting that the geological template of the Cretaceous rift directly influences the flow of Thwaites Glacier. We suggest that this influence is exerted via faults juxtaposing different lithologies at the ice-bed interface, unmediated by sedimentary cover.

Our study suggests that granitic intrusions form several of the subglacial highlands in the Thwaites Glacier catchment (Fig. 6). These highlands steer ice flow, for example, separating Thwaites and Pine Island Glaciers. Understanding the location of these granite bodies is important as they likely formed durable highlands around which past glaciers, ice streams, and preglacial river systems would have flowed. Hence, understanding the distribution of this lithology is important for predicting the long-term landscape evolution of this sector of West Antarctica.

### Implications for subglacial water

The restricted nature of the sedimentary basins we interpret has implications for the distribution and flow of subglacial water beneath Thwaites Glacier. Subglacial aquifers can theoretically accommodate large volumes of ground water flow, changing water pressure at the bed, and hence influencing basal friction (*11*). However, the apparent lack of a thick sedimentary basin beneath ~80% of the faster flowing Thwaites Glacier catchment may mean that more water is present at the ice-bed interface here than in other catchments, such as the Siple Coast, where a groundwater reservoir may hold up to 45% of the basal water (*10*). The impact of widespread impermeable bedrock, rather than a semipermeable sedimentary substrate, on the style and efficiency of water flow through either distributed or channelized systems will be an important factor to quantify for future studies of Thwaites Glacier stability.

### Implications for geothermal flux

The apparent dominance of Cretaceous rifting on geological structures within the Thwaites Glacier catchment likely influences the region’s geothermal heat flux. During rifting, thinning of the lithosphere draws hot asthenospheric mantle toward the surface, increasing the geothermal gradient and enhancing surface heat flux (*52*). However, this effect is transient because the mantle cools over the subsequent 60 to 100 Ma. In addition, almost 50% of the surface heat flux in continental regions is due to radiogenic decay within the crust so that stretching and thinning of the crust by rifting can lead to a permanent reduction in geothermal heat production (*53*). Combining these two factors suggests that the heat flux over a Cretaceous rift may be relatively low. As we interpret the geological features in the Thwaites Glacier region to be generally associated with Cretaceous rather than more recent rifting, regionally high heat flux beneath the glacier should not automatically be assumed. Factors such as rejuvenation by upwelling hot mantle, as seismically imaged beneath MBL (*25*), potential Cainozoic extension (*22*), or highly radiogenic granites (*54*) may increase geothermal heat flux, but must all be considered in the context of a likely cool Cretaceous rift.

### Future work

Our interpretation and discussion sets the stage for future investigations into the coupling between basal conditions and ice flow. A priority is characterizing the variations in basal friction between areas where sedimentary basins are present and absent, especially under relatively fast flowing ice. Such investigations along the trunk of Thwaites Glacier, from the ridge where it is currently stabilized, extending upstream across the area of reverse slope to the next pinning ridge are key. Characterizing the detailed variability in basal friction in this area, where we have constrained the geology, will allow better understanding of how the geology will influence the basal shear stress in upstream areas as the ice sheet retreats and the driving stresses evolve. An additional hypothesis, suggested by our interpreted map (Fig. 6), is that ground water flow may be restricted because of thin/absent sediments in many parts of the Thwaites Glacier catchment. This can be tested by, for example, magnetotelluric studies (*55*) and is important as movement of water in and out of subglacial aquifers may play an important but poorly understood role in glacial basal hydrology and lubrication of ice flow, as well as mediating transfer of geothermal heat. Magnetotelluric studies would also provide a useful and independent estimate of the thickness of the interpreted sedimentary basins, which in this study are only loosely constrained by simplified considerations of gravity anomaly amplitude.

By revealing the likely extent of Cretaceous rift–related magmatism, our study confirms the separation of Antarctica and Zealandia as a highly magmatic event. Future geodynamic modeling can use this information to constrain the processes driving continental separation in this region. Further investigation into why the interpreted pattern of rift parallel magmatism appears to be restricted to the Thwaites Glacier region is required to fully characterize this sector of the West Antarctic Rift System. We hypothesize that the along-strike change toward Pine Island Glacier is a primary feature reflecting a major reduction in magmatism in this direction. In contrast, we propose that the apparent change in magnetic trends and interpreted magmatism toward Mt. Murphy and Mt. Takahe reflects later overprinting of the rifted margin signatures associated with the development of the MBL Dome.

## MATERIALS AND METHODS

Thwaites Glacier has been a focus of scientific study for almost 2 decades (fig. S1). Our objective in this study is to create a series of geophysical maps and models unbiased by regional trends, or survey layout, enabling the underlying geology to be interpreted. Two aerogeophysical surveys called AGASEA and BBAS underpin our study, providing regional airborne magnetic, gravity, and radar data (*56*, *57*). NASA Operation Ice Bridge (OIB) provides detailed airborne radar and gravity data up to 70 km inboard of the grounding line. The International Thwaites Glacier Collaboration (ITGC) 2018–2020 seasons add magnetic, gravity, and radar data across the glacier up to 195 km from the grounding line. Offshore helicopter aeromagnetic surveys provide regional context for our interpretation (*20*). For details on the surveys used in this study, see text S1 and table S1. The various datasets are processed together to provide geophysical observations and model outputs that inform our interpretation (fig. S2A).

### Topographic compilation and bed roughness

Topography beneath the ice sheet provides a first-order view of the geomorphology and is influenced by the underlying geology. Removing the topographic signature is also a vital step in processing airborne gravity data. For geomorphology and gravity processing, direct radar observations of bed elevation are preferred, as extrapolation based on ice sheet flow models may introduce artifacts (*58*). We therefore compiled the along-track radar-derived bed elevation picks from the surveys in table S1, which were interpolated onto a 2-km mesh raster. Bathymetry beneath the surrounding ice shelves was derived from gravity data, with swath bathymetry used further offshore (*59*). Beyond these datasets, BEDMAP2 provides a regionally complete grid (Fig. 2A). Subglacial topographic roughness was calculated as the along-track standard deviation of the picked radar bed elevation within a ~1-km-wide moving window. The reported standard deviation values exclude areas where the bed elevation was poorly resolved, defined as having along-line data gaps of >500 m, i.e., >50% of the analysis window. Standard deviation provides a good first-order assessment of rough and smooth regions of the bed (*60*). Calculated on-line bed roughness was interpolated onto a 2-km mesh raster for display and interpretation (Fig. 2B).

### Aeromagnetic compilation and magnetic source locations

The aeromagnetic compilation included line data available through the ADMAP2 project (*61*) and more recent ITGC data (see text S1). Combination of all line data into a single database, reassessment of leveling errors, and regridding ensure that anomalies at survey boundaries are accurately reproduced and line noise is minimized. Line data were continued to 500 m above the ice surface, giving a consistent observation elevation while maintaining maximum short wavelength information (fig. S3A). After leveling and continuation, crossover errors were 18.79 nT. An alternative continuation scheme to 2500 m above the bed topography, giving a constant minimum depth to source across the survey, was tested (fig. S3B) but makes no difference to the regional interpretation. For display, we interpolated the line data, continued to 500 m above the ice surface, onto a 1-km mesh. This was reduced to the pole (*62*), theoretically aligning anomalies over their sources (Fig. 2C).

The edges of magnetic sources were located using both tilt angle and TDX enhancements calculated from the gradients of the reduced-to-the-pole magnetic field (*63*, *64*). Assuming that sources have vertical sides and no remnant magnetization, zero tilt angle and maximum TDX values locate source edges. This assumption produces errors in location proportional to the true dip of the contact and its depth (*65*). Given that depth-to-source analysis suggests that most magnetic sources are less than 5 km deep, errors in the lateral location of source body margins on the order of 5 km are expected, which is small relative to the size of the study area. To define the trends in the magnetic data, the length and direction of tilt angle zero contour line segments within a set of 125-km^2^ windows were plotted as rose diagrams (fig. S4). A 30-km low-pass filter before extraction of the tilt angle zero contour helped isolate the dominant trends.

### Magnetic depth-to-source calculation

Magnetic source depths (Fig. 3) were estimated from gridded data using the tilt depth and 3D extended Euler deconvolution methods (*64*, *66*). The gridded magnetic data, continued to 500 m above the ice surface, were the input. The tilt depth method assumes that the source is a vertical contact (structural index of zero) traced by the tilt angle 0° contour and that the depth is proportional to the distance from the contact to the adjacent positive or negative tilt angle contours (*64*). Low-confidence solutions were identified and excluded in areas where the depth estimates from the adjacent positive and negative contours did not agree, for example, because of complex contour geometry resulting from high-frequency anomaly patterns (see text S3 for details). The selected valid points had a standard deviation of 780 m between the depth estimated from the positive and negative tilt angle contour, which is taken as the error of the depth estimates. The 3D extended Euler method was implemented using software from the U.S. Geological Survey (*66*). We assumed a structural index of zero and an analysis window 11 km wide. The extended Euler method returns up to seven solutions for each analysis window, which are averaged to provide the final source location, while the range of the solutions is used to isolate valid results (*66*). We chose to include results with an error in depth between estimates of <5% and more than four valid solutions.

Depth-to-source solutions along profiles crossing key coastal magnetic anomalies were calculated (Fig. 4). The input data were the along-line magnetic field after continuation to 2500 m above the bed. Methods were applied using the Geosoft software suite and included analytical signal, Werner, and Extended Euler. Analytical signal and Werner methods used 3- to 5-km-wide windows, while extended Euler solutions assume a ~5-km window. These window widths are sensitive to relatively shallow sources, most likely to approach the ice-bed interface and influence ice flow. Structural index values of 0 (contact) or 1 (dike) were considered as the potential source geometry. These assumptions and noise in the data distort the results, meaning that individual solutions are not robust and errors are hard to statistically quantify; however, solution clusters form a guide to the true magnetic source location (*67*).

### Gravity compilation and reduction

We constructed a regional gravity compilation using line free-air anomaly data from the surveys noted in table S1. The input datasets required detailed editing and leveling to ensure a consistent and high-quality product free of apparent artifacts. A single database including all available line free-air gravity data was constructed. We excluded data collected at anomalously high altitudes, or associated with changes in flight altitude, which gave rise to line noise in gridded products. Statistical leveling, with the OIB dataset as a fixed reference, further reduced line-to-line noise. Last, the free-air anomaly data were continued to a uniform level of 2500 m (fig. S5A). After leveling and continuation, the line free-air data had an internal crossover error of 2.94 mgal.

The signature of the subglacial topography dominates the free-air gravity anomaly (fig. S5A). The gravity effect of the subglacial topography, ice sheet, and offshore bathymetry calculated from our topographic compilation is the Bouguer correction, for which we assumed standard densities of 2670, 915, and 1028 kgm^−3^ for rock, ice, and water, respectively. Where the density of the topography differs from the standard, gravity anomalies correlated with the topography will be retained in the Bouguer anomaly. These retained anomalies will be proportional to any discrepancy in density, allowing the effects to be correctly considered in subsequent modeling. We note that the topography beneath the ice shelves is based on inversion of gravity data, and interpretation of Bouguer and derived gravity anomalies over the ice shelves is not valid. However, our study area is onshore, and use of gravity-derived topography in adjacent areas, required to create a seamless Bouguer correction grid, will not distort the shape or amplitude of the onshore anomalies.

The Bouguer gravity anomaly map (fig. S5B) shows little short wavelength resemblance to the subglacial topography, indicating that local topographic effects have been well modeled. However, isostatic compensation of the ice and topographic loads by low-density material at depth creates the observed long wavelength Bouguer gravity anomalies anticorrelated with the topography. The gravity effect of this compensation is modeled assuming Airy isostatic balance and subtracted from the Bouguer gravity anomaly to give the final residual Airy isostatic anomaly. The Airy isostatic model assumes no lateral strength in the crust and that the modeled gravity anomaly results from deflection of the Moho beneath elevated topography about a fixed compensation depth of 29 km. Such a model is not intended to be a true and robust reflection of crustal thickness, or the compensation method; rather, it is intended to quickly capture the gravity effect of the density variations required to support the observed topography in a simple and repeatable manner. The derived residual Airy isostatic anomaly is theoretically due only to upper crustal density variations (Fig. 2D). For details on airborne gravity compilation and corrections, see text S4.

### 2D modeling

To test the hypothesis that bodies with both elevated density and elevated magnetic susceptibility can cause the spatially correlated anomalies beneath the trunk of Thwaites Glacier, we constructed 2D forward models of the residual Airy isostatic gravity and magnetic anomalies (Fig. 4). This composite profile was chosen as it is orthogonal to the main anomalies across the mouth of Thwaites Glacier. Additional models along adjacent flight lines (fig. S6) support the result on the central profile. Such 2D models are nonunique; however, they allow exploration of the extent, geometry, and properties of the source bodies. Magnetic depth-to-source solutions provide a loose constraint on the forward models. The background density was set to 2670 kg m^−3^ as the Bouguer correction used this value and is assumed to account for density contrasts at and above the ice-bed interface. Where the assumed Bouguer correction density is incorrect, topography will be associated with local residual gravity anomalies, which can be appropriately modeled. The Airy isostatic correction is assumed to account for gravity anomalies associated with variations in crustal thickness or other regional isostatic compensation mechanisms. Variations in Moho depth or mantle density are therefore not considered in our 2D models as their signatures have theoretically been removed. The lack of active source seismic constraints means that additional mid to lower crustal sources for the observed magnetic and gravity anomalies, or complex upper crustal geometries, cannot be justified. In addition, estimates of Curie depth in this region of between 10 and 20 km suggest that few mid to lower crustal magnetic sources are present (*9*), further justifying only modeling magnetic sources within the upper crust.

### 3D inversion

An alternative to manual forward modeling is 3D inversion, which recovers an optimum, but nonunique, 3D distribution of magnetic susceptibility or rock density giving modeled anomalies matching those observed. We used the VOXI 3D inversion module of the Geosoft software suite (*68*). Density or susceptibility values are assigned to each cell (voxel) in a 3D mesh and are iteratively varied until a stable solution fitting the input data has been achieved. No robust constraints on the location of the sources, such as seismic sections or bore-hole data, are available in this region, limiting the ability of the inversion to produce definitive solutions for source location or rock properties. However, constraints on the maximum range of the recovered rock properties, along with the model dimensions and boundary conditions, direct the inversion toward a geologically reasonable result, providing an initial 3D estimate of source geometry and volume beneath the trunk of Thwaites Glacier (Fig. 5). For details of the 3D inversion, see text S5.

## Supplementary Materials

### This PDF file includes:

Supplementary Text Figs. S1 to S11 Table S1 References
