# Supplementary material for: Geological sketch map and implications for ice flow of Thwaites Glacier, West Antarctica, from integrated aerogeophysical observations
Source: Sci Adv. 2023 May 31;9(22):eadf2639. doi: 10.1126/sciadv.adf2639 (PMC10413667; doi:10.1126/sciadv.adf2639)
Supplement: Supplementary file 1 — Supplementary Text Figs. S1 to S11 Table S1 References [file sciadv.adf2639_sm.pdf]

Science Advances
AAAS

Supplementary Materials for

Geological sketch map and implications for ice flow of Thwaites Glacier, West Antarctica, from integrated aerogeophysical observations

Tom A. Jordan *et al.*

Corresponding author: Tom A. Jordan, tomj@bas.ac.uk

Sci. Adv. **9**, eadf2639 (2023)
DOI: 10.1126/sciadv.adf2639

This PDF file includes:

Supplementary Text
Figs. S1 to S11
Table S1
References

Supplementary Text

S1. Geophysical surveys over the Thwaites Glacier catchment.

The first systematic surveys of the Thwaites Glacier region were the UK-US collaborative AGASEA and BBAS aerogeophysical surveys in the 2004/05 field season (Fig. S1). These surveys achieved a line spacing of 15 and 30 km for the Thwaites and Pine Island catchments respectively. The surveys collected radar depth-sounding (*56, 57*), gravity (*23, 29*) and magnetic data (*7, 61*), and underpin our study. NASA Operation Ice Bridge (OIB) airborne missions between 2009 and 2019 collected radar depth-sounding (*71*) and gravity data (*72*), but no magnetic data. OIB missions focused on the down-stream part of Thwaites Glacier, defined here as <70 km from the grounding line. Data was collected with 2.5 km line spacing up to 45 km inland and 5 km line spacing data up to 70 km inland. Additional OIB transects approximately 30 km apart, orientated orthogonal to ice flow, covered up-stream areas. The International Thwaites Glacier Collaboration (ITGC) collected additional airborne radar, magnetic (*73, 74*) and gravity data (*75, 76*) in the Thwaites Glacier region in the 2018/19 and 2019/20 field seasons, augmenting earlier surveys. This includes providing areas of ~5 km line spacing magnetic data in the down-stream sector, and extending the region with 2.5 km line spaced radar and gravity data from 45 to 70 km inland. In the central sector, 70 – 195 km from the grounding line, the ITGC data interleaves the AGASEA survey, reducing the effective line spacing from 15 km to 7.5 km. Offshore, helicopter and ship-borne data extends the coverage of the aeromagnetic data set across the continental shelf (*20*).

S2. Aeromagnetic compilation

The aeromagnetic compilation was created from line data made available through the ADMAP2 project (*61*) and incorporating more recent ITGC data (*73, 74*). All data was merged into a single data-base and levelled using the ADMAP line data as the initial reference. Combination of all line data into a single database and re-gridding ensures anomalies at survey boundaries are accurately reproduced. The ITGC data underwent standard processing to account for the global reference field, errors introduced by operational procedures and diurnal variations, before it was merged and statistically levelled to the older datasets. Visual inspection of the resulting grid revealed some areas where additional levelling, or removal of inconsistent data was required. This most importantly included removal of all data collected in the 1970s by SPRI, which in this region is superseded by more modern and better geo-located data. Data were upward/downward continued to 500 m above the ice/sea surface to place all observations on a common surface (Fig. S3a). This continuation level was chosen as it preserves high frequency anomalies, while minimizing the extent that data is downward continued. An alternative continuation to 2500 m above the underlying rock was also carried out to give a view with a consistent distance to the closest possible source (Fig. S3b). The final continued line data were interpolated onto a 1 km mesh and reduced-to-the-pole (Fig. 2C), a procedure which theoretically aligns anomalies with their sources.

S3 Tilt depth magnetic depth to source method

For the tilt depth method the tilt angle was first calculated from the horizontal and vertical gradients of the magnetic field (*77*). The distance from every node on the zero tilt angle contour to the nearest +30° and -30° tilt angle contour was then extracted using ArcGIS ‘near’ function. The distance to the up and down contour were transformed to estimates of source depth and averaged (*64, 78*). Two tests were then applied to isolate robust depth solutions, assuming the distance to the positive and negative tilt contour are independent estimates of source depth. First,

the vectors to the closest positive and negative contours were within ±30° of being diametrically opposite. This prevents local kinks and areas with complex contours biasing the result. Second, points where depth estimates from the positive and negative contours differed by more than 50% of the average depth were excluded.

S4. Airborne gravity compilation and corrections.

Line gravity data from the surveys noted above was used to construct the free air gravity grid for this study. Detailed data editing and levelling was carried out prior to gridding to ensure a consistent and high quality product free of apparent artefacts. OIB data collected >1500 m above the ice surface was discarded, as the lack of resolution of short wavelength features on these transit flights distorts the resulting integrated gravity field. The line data from ITGC, AGASEA and BBAS were initially statistically levelled using the OIB dataset as a reference. Subsequent additional statistical levelling was applied to AGASEA, ITGC, and BBAS datasets to improve the gridded product. This included in places editing the AGASEA line data where erroneous anomalies became apparent on gridding. These regions typically corresponded to areas of aircraft elevation change where the L&R gravity system is known to perform poorly. Prior to gridding the line free air data was upward, or in some cases downward continued to a uniform level of 2500 m. This elevation was chosen as it is above the flight altitude of 95% of the dataset, minimizing the need for application of downward continuation, which is inherently less robust (Fig. S5a).

The full 3D gravity effect of the topography, bathymetry and ice was accounted for by calculating the Bouguer correction. For this the 2km mesh raster of topography was used as an input, including data up to 150 km from our study area to mitigate any lateral terrain effects. Ice surface elevations were taken from the BEDMAP2 data compilation (*79*). An observation altitude of 2500 m was used, as it is above the surface elevation across most of the study area. This is not true for the peak of Mt Takahea and some other of the volcanic peaks which lie outside our study region, but within the region of calculation. These discrepancies have limited impact on the calculated correction grid, but anomalies over Mt Takahea cannot be interpreted. Use of an observation level above all topography (~4000 m) would require additional continuation, degrading the observed gravity signal in the areas of most interest. We also note that the Bouguer correction is not robust over the floating ice shelves, as the bathymetry is initially derived from gravity data.

The Bouguer correction was calculated using a 3-D Gauss-Legendre quadrature (GLQ) method (*80*), which calculates the gravity effect of prisms of material with a specific density bounded by an upper and lower surface at each point on an observational mesh. The gravity effect of four layers was considered. Ice (915 kgm^{-3}), water (1028 kgm^{-3}), topography above sea level (2670 kgm^{-3}) and topography below sea level (-2670 kgm^{-3}). Summation of the gravity effect of these four layers provides the Bouguer correction. This correction was sampled onto the flight lines and subtracted from the free air anomaly. The resulting Bouguer anomaly was interpolated onto a 2 km mesh raster. The Bouguer gravity anomaly grid was filtered with 3 passes of a 9x9 Hanning filter to minimize residual line to line noise (Fig. S5b).

The final correction applied to the gravity data aims to isolate gravity anomalies associated with crustal geology from the gravity effect of isostatic compensation of the surface topographic, ice and water loads. Isostatic compensation is typically associated with a buoyant low-density crustal root beneath elevated topography. A simple way to calculate the magnitude of the compensating crustal root is to assume that the surface loads are compensated locally, i.e. by a buoyant root directly below. Such Airy compensation is consistent with the low elastic thickness calculated by previous studies in the adjacent Pine Island Glacier region (*23*). In the Thwaites Glacier region, especially towards Mary Byrd Land, the topography may be supported by the buoyancy of warm mantle, rather than a crustal root, i.e. so called Pratt type isostatic support (*27*).

However, we argue that an Airy model will adequately remove the first order gravity effect of isostatic support of the topography. The long wavelength gravity effect of the low density buoyant mantle required to support the topography will be approximately equivalent to the effect of a low density crustal root, as the cumulative mass deficiency required to support the topography is the same. The size and gravity effect of the compensating root was calculated using a fast Fourier transform routine within the GMT software package, assuming a uniform crustal density of 2670 kgm^{-3} and a mantle density of 3330 kgm^{-3}. This calculation requires assumption of a reference depth for the mantle where the surface topography has zero elevation. We chose a reference depth of 29 km, as this gives a mean predicted Moho depth matching the mean result from passive seismic estimates in this area (*81*). The Airy isostatic gravity model was subtracted from the Bouguer anomaly to give the final residual Airy isostatic anomaly (Fig. 2D).

S5. Detail of 3D inversion inputs and parameters.

The 3D inversion used the VOXI 3D inversion module of the Geosoft software suite (*68*). The inversion predicts a three-dimensional distribution of susceptibility or density from input magnetic or gravity observations. It does this using a Tikhonov minimum gradient regularisation (*82*) to produce a model conforming to a set of reference Earth properties, where the modelled response closely matches the input data. The inversion included a proprietary iterative reweighting inversion (IRI) focusing function, which acts to sharpen the boundaries of source bodies. By limiting the user-intervention to global parameters such as the minimum/maximum values for rock properties and depth weighting, use of this inversion produces an output for interpretation relatively unbiased by user intervention. However, we acknowledge that the inversion output, like other minimally constrained potential field models, is non-unique and other geometries could provide an equivalent fit to the data. The input magnetic field for the 3D inversion was the magnetic anomaly continued to 500 m above the ice surface, with a mean and best fit linear trend removed. Susceptibility values were constrained to be >0 and $<75\times10^{-3}$ SI consistent with values for typical igneous rocks (*38*). The recovered source properties implicitly include the impact of any magnetic remanence and are therefore apparent susceptibility values. An active magnetic model volume extending from 0 km to -10 km depth, with a mesh resolution of 1.25/1.25/0.5 km was used, with an expansion ratio of 1.5 with depth. Five padding cells were included below the active model. As many of the depth to source solutions indicate shallow sources, and to counter the tendency of the inversion to assign higher susceptibility values to deeper levels in the model, we imposed an additional linear weighting scheme favoring higher susceptibility values at shallower depths.

When inverting for density the residual Airy isostatic gravity anomaly, with a mean and best fit linear trend removed was considered as the input data. Density was constrained to lie between +/-1000 kgm^{-3}, and recovered values reflect a density contrast, rather than absolute density values. An active model volume extending from ~2.5 km to -12 km depth, with a mesh resolution of 2.5/2.5/1.25 km was used, with an expansion ratio of 1.5 with depth. Five padding cells below the active model were included. Finer mesh resolution, or weighting the results to favor shallow results failed to give valid results within the inversion scheme.

For the magnetic and gravity inversion cells within the ice column were set to either zero susceptibility or zero density contrast, ensuring recovered geophysical sources were below the ice-bed interface.

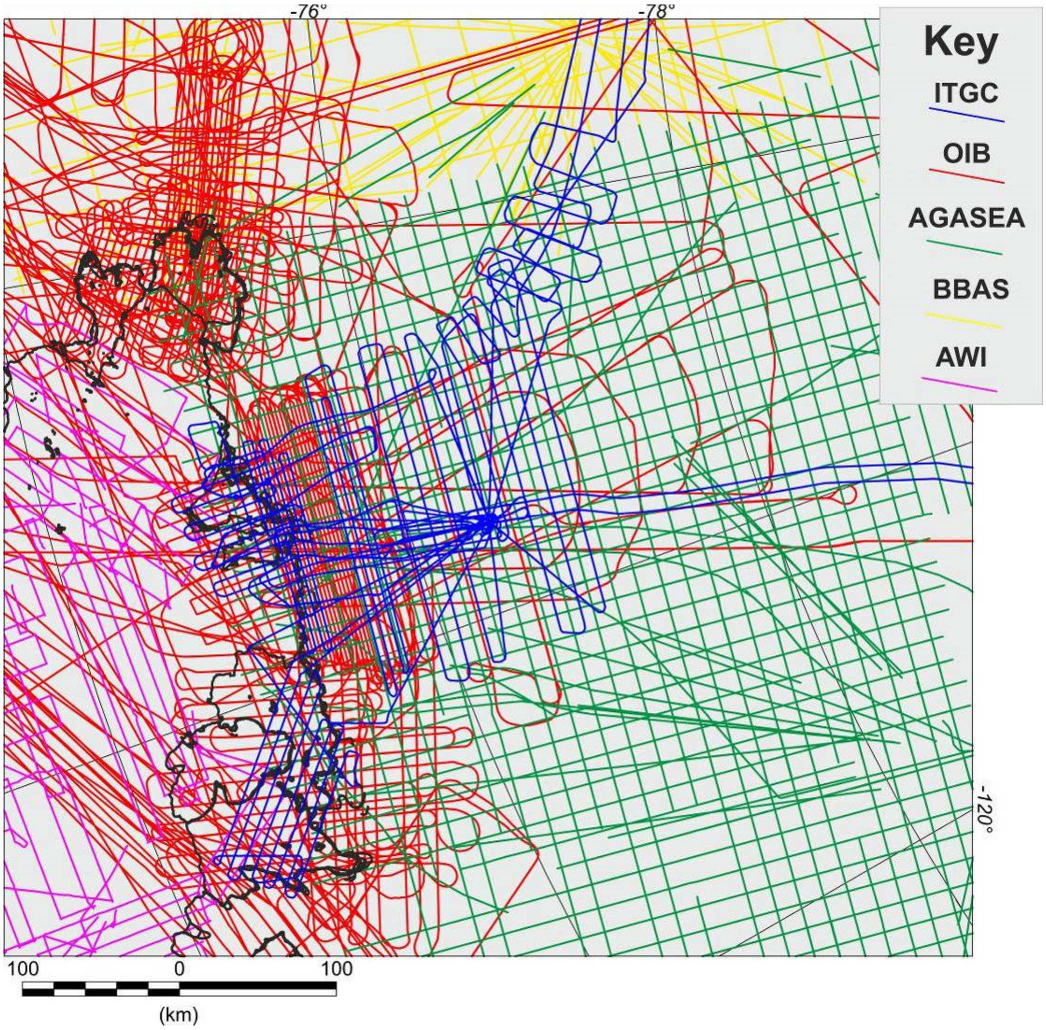

Fig. S1.

Location of aerogeophysical data. Black-line locates coast and encloses floating ice shelves. The International Thwaites Glacier Collaboration (ITGC), BBAS and AGASEA surveys collected radar, magnetic and gravity data. Operation Ice Bridge (OIB) dataset does not include magnetic data, while the offshore Alfred Wegener Institute (AWI) survey only includes magnetic data (See Table S1).

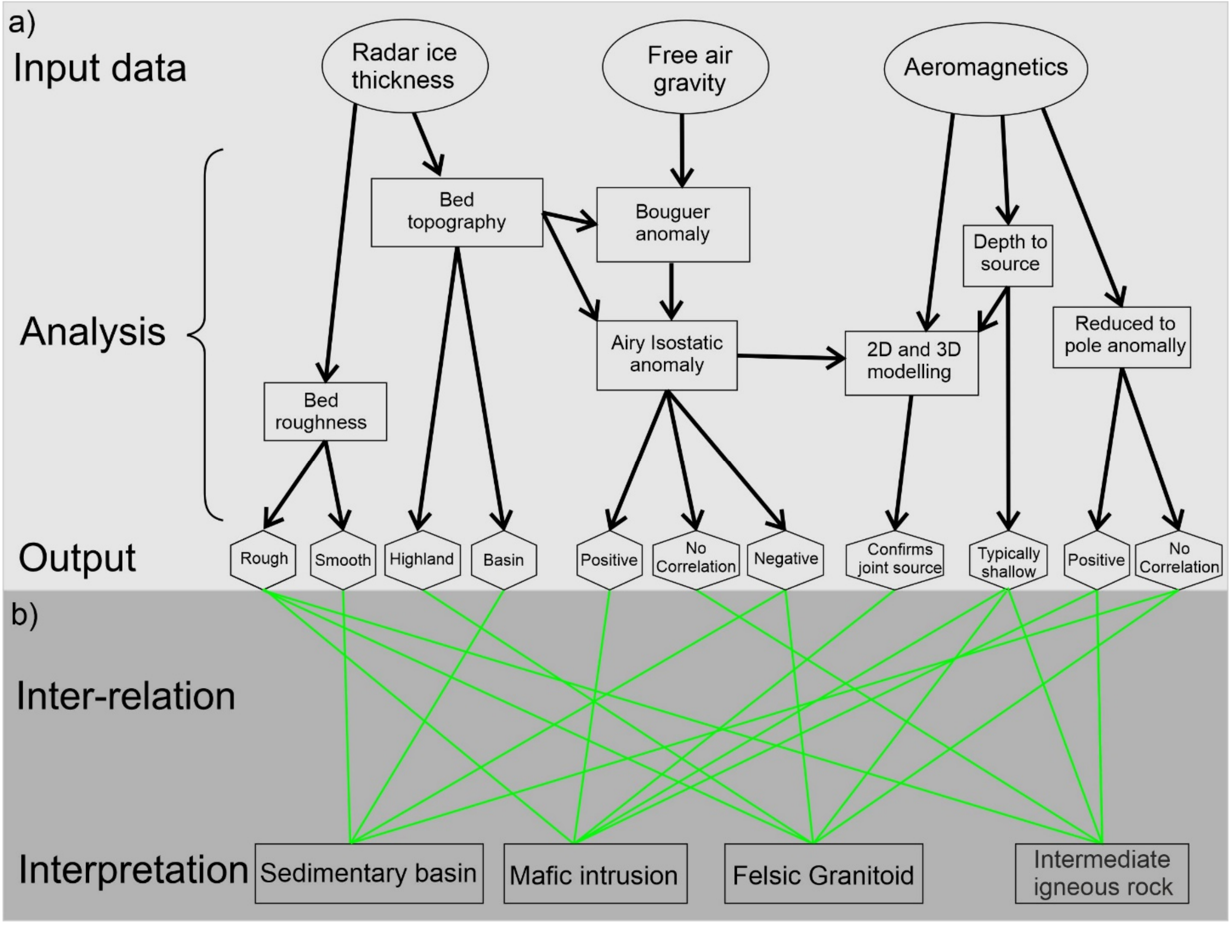

Fig. S2.

Flow chart from input data to interpreted geological sketch map. a) Data processing and analysis section showing interdependency of datasets with output geophysical observations and models. b) Visual schematic showing how the different results of the geophysical observations and analysis help build our final geological interpretation of each lithology. Note for Airy isostatic gravity and magnetic anomalies "No Correlation" in the Output row means neither positive nor negative anomalies, nor trends associated with these observations are clearly correlated with the other geophysical features of the interpreted lithology.

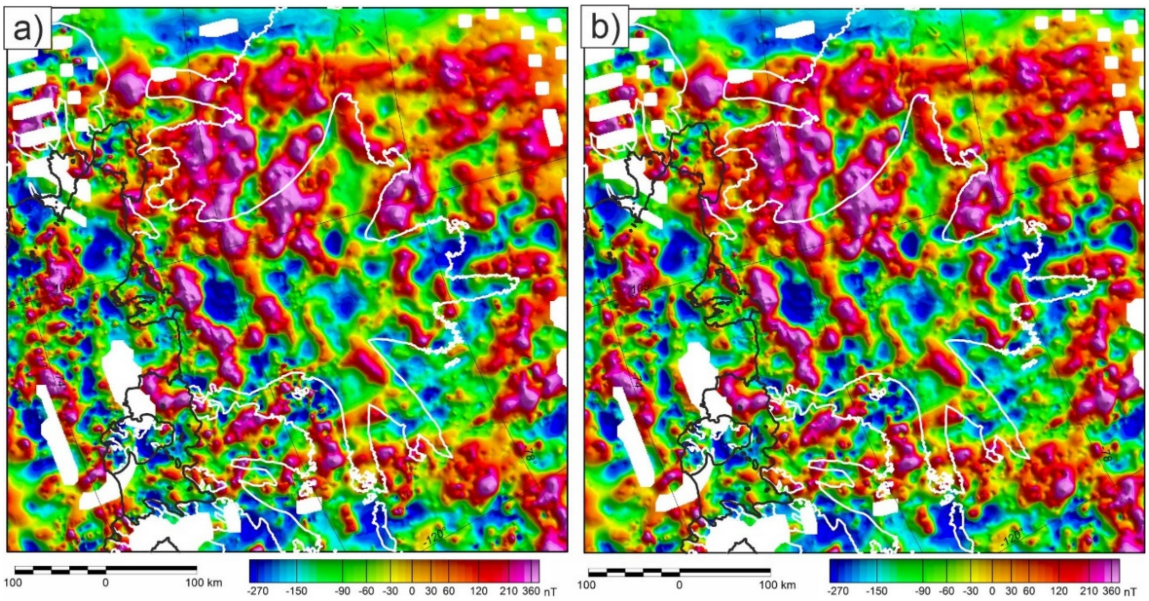

Fig. S3.

Processed aeromagnetic data with alternative continuation methods. Black line marks coast and white line locates 40 ma^{-1} ice velocity contour. a) Data continued to 500 m above the ice surface, retaining maximum resolution. b) Data continued to 2500 m above the bed. Note slight suppression of shorter wavelength anomalies in offshore regions with the second continuation method.

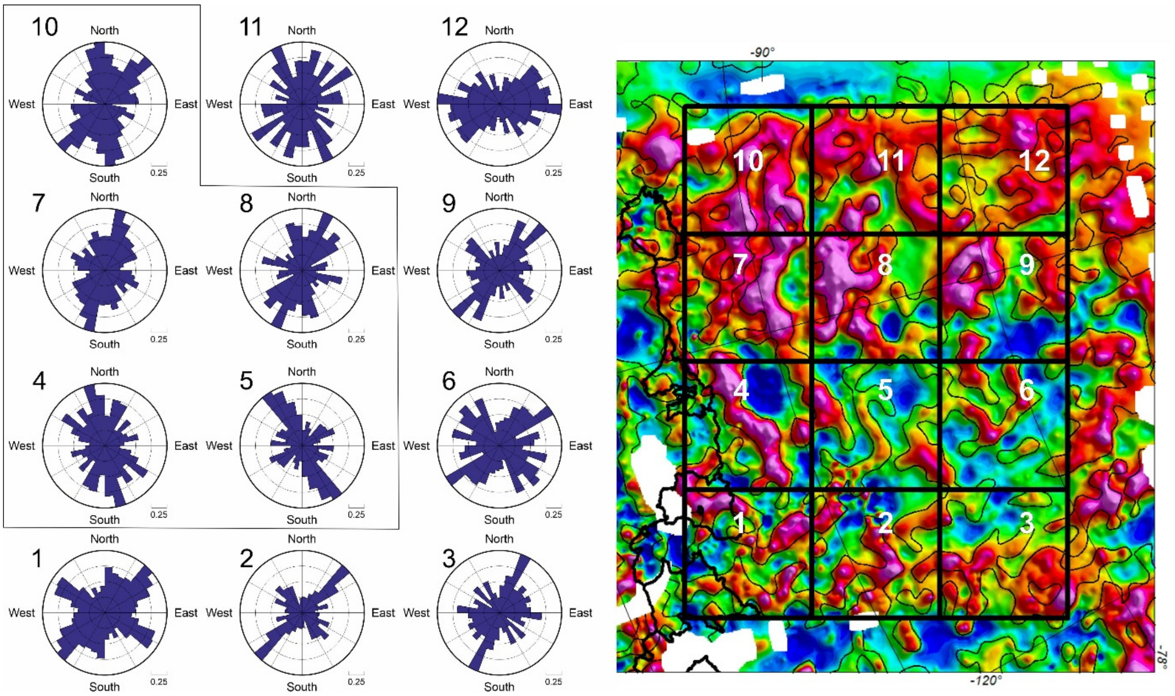

Fig. S4.

Rose diagrams showing magnetic trend orientation in 125 km square windows distributed across the study area based on analysis of the tilt angle zero contour (thin black line on map). Magnetic trend orientations are referenced to the projected map orientation, not true geographic directions. Note areas 4,5,7,8, and 10 show a broadly grid N-S trend, while other areas show more complex trends, with a tendency to a grid NE-SW trend.

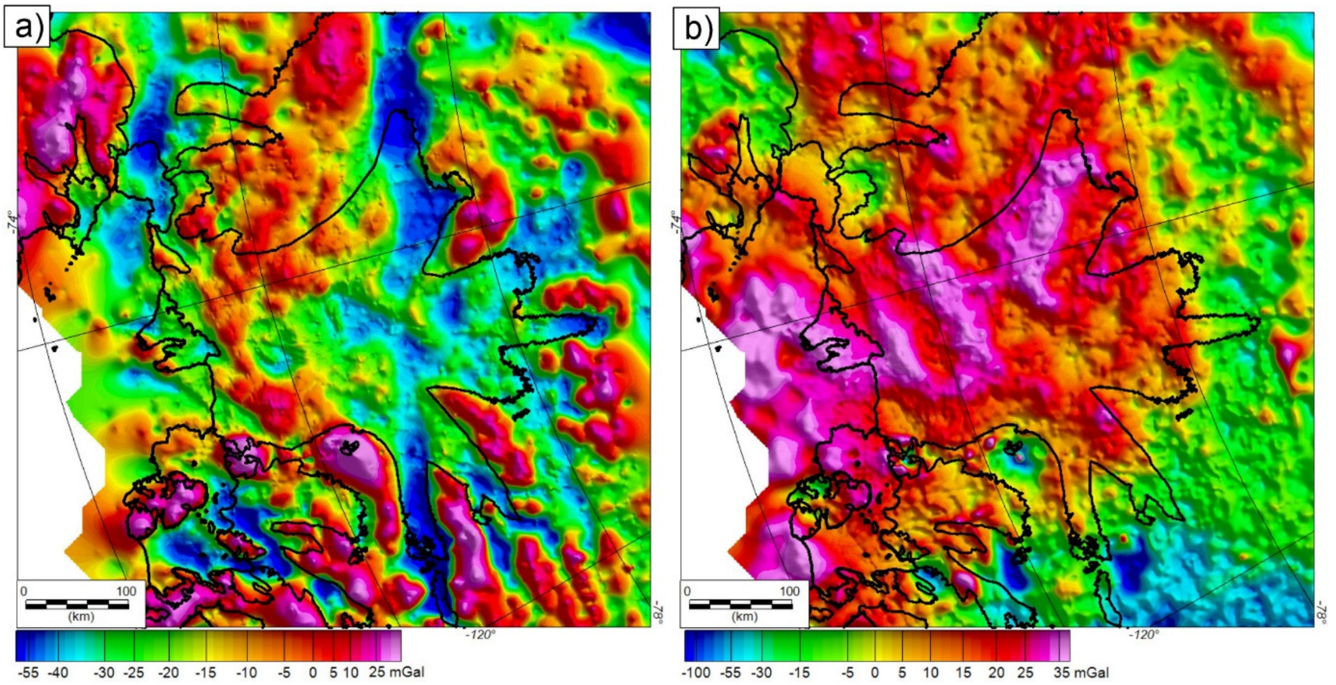

Fig. S5.

Gravity anomaly compilation maps. Black lines mark coast and 40 ma^{-1} ice velocity contour. a) Free air gravity anomaly continued to 2500 m elevation. b) Bouguer gravity anomaly map with three pass Hanning filter to remove residual line noise.

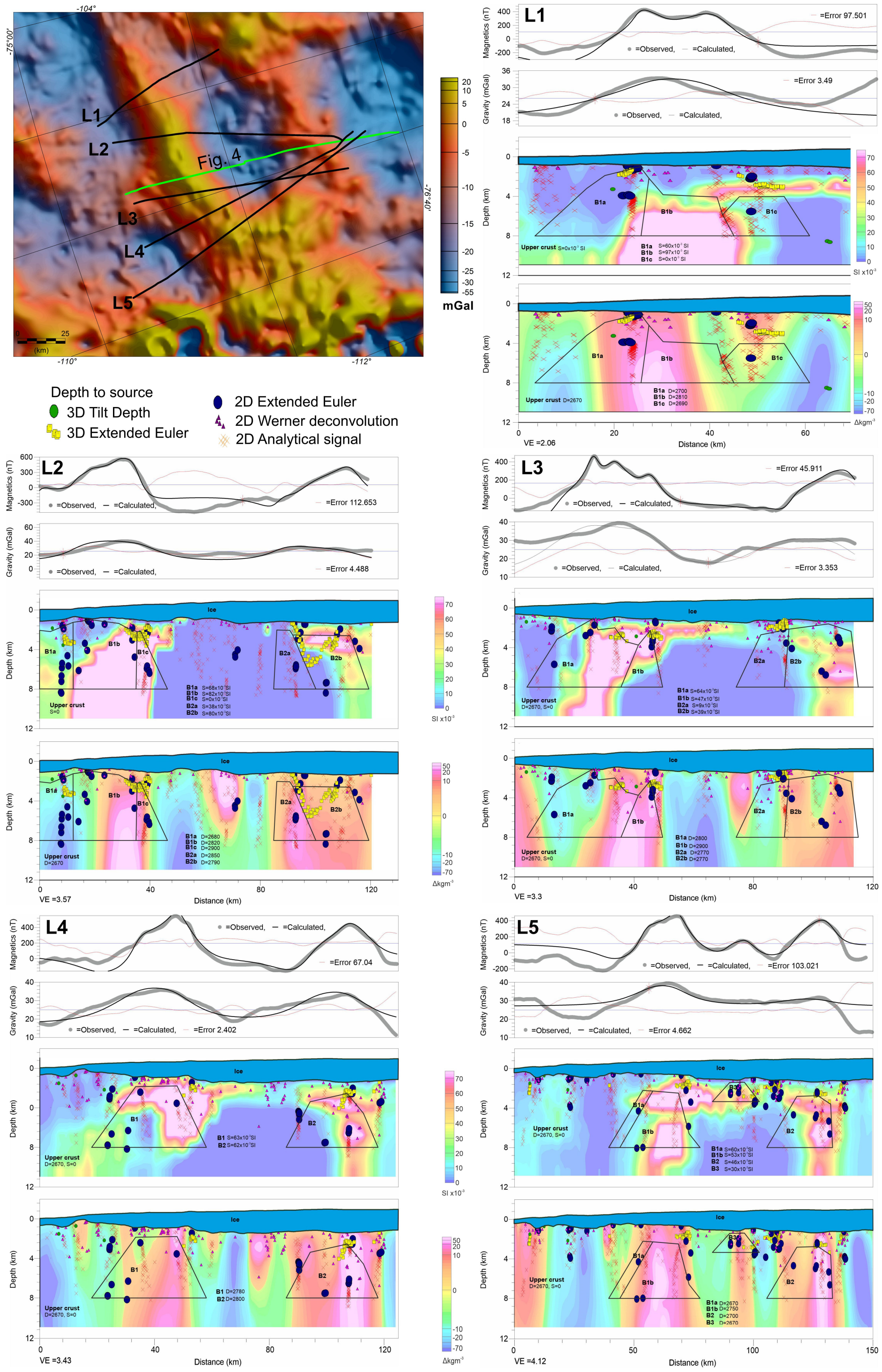

Fig. S6.

Additional 2D forward models and slices of 3D inversion volume over down-stream magnetic and gravity anomalies. Map shows Airy isostatic anomaly and locates profiles L1 to L5, and the key profile K-K' shown in the main text Fig. 4. For each profile upper panels show observed and forward calculated magnetic and gravity anomalies while lower panels show 2D forward models (black bodies) and inverted magnetic susceptibility or density structure.

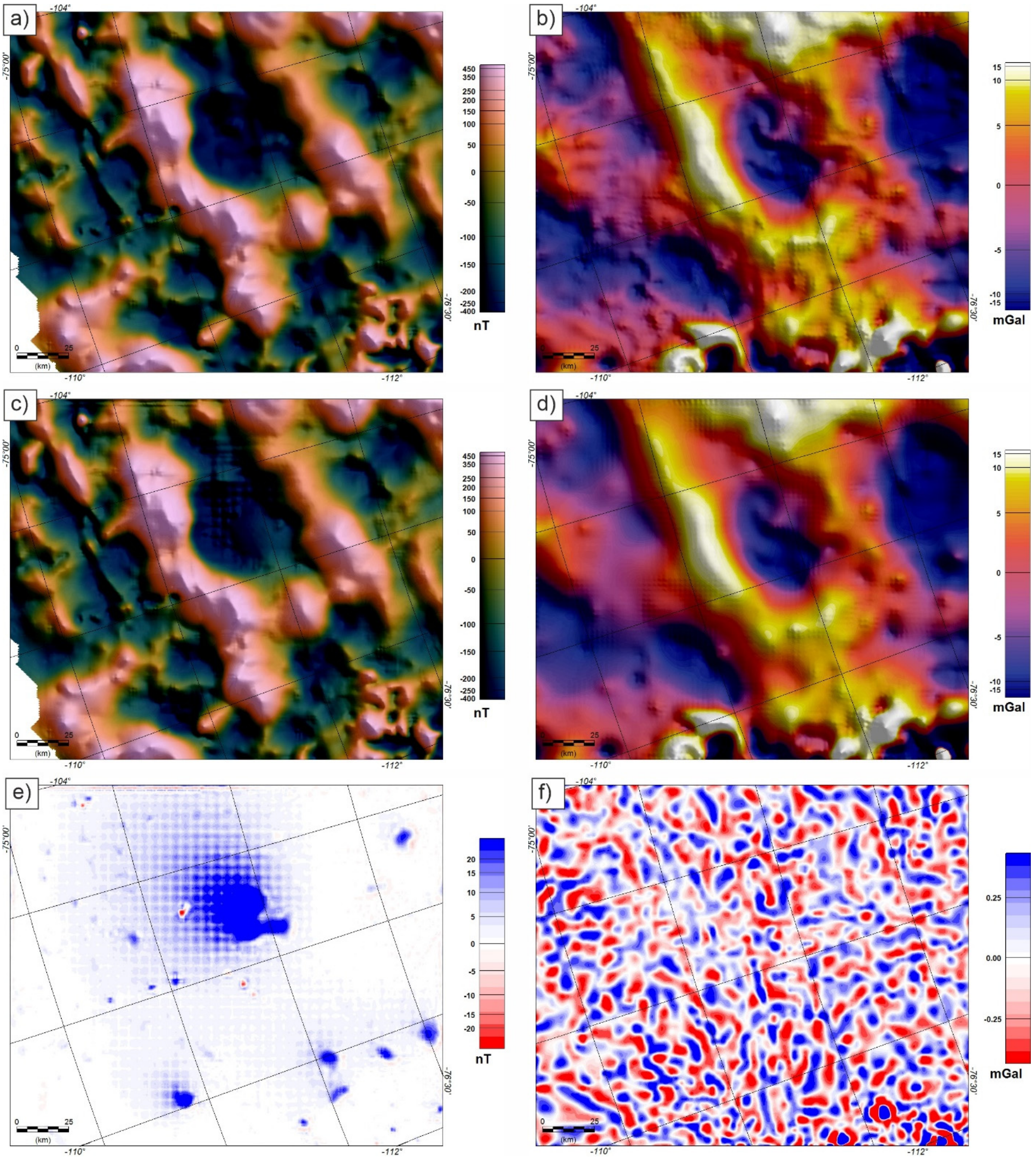

Fig. S7.

Input and output data, together with difference maps for the 3D inversion. a) Input magnetic anomaly grid. b) Input Airy isostatic gravity residual grid. c) Predicted magnetic anomaly map. d) Predicted gravity anomaly map. e) Magnetic anomaly error after inversion (input – output). Overall standard deviation is 8.5 nT, but a negative of up to -120 nT is present indicating magnetic remanence may be an issue in this area. f) Gravity anomaly error after inversion (input – output). Overall standard deviation is 0.35 mGal, suggesting the model is over-fit compared to the quality of the input data (2.94 mGal standard deviation).

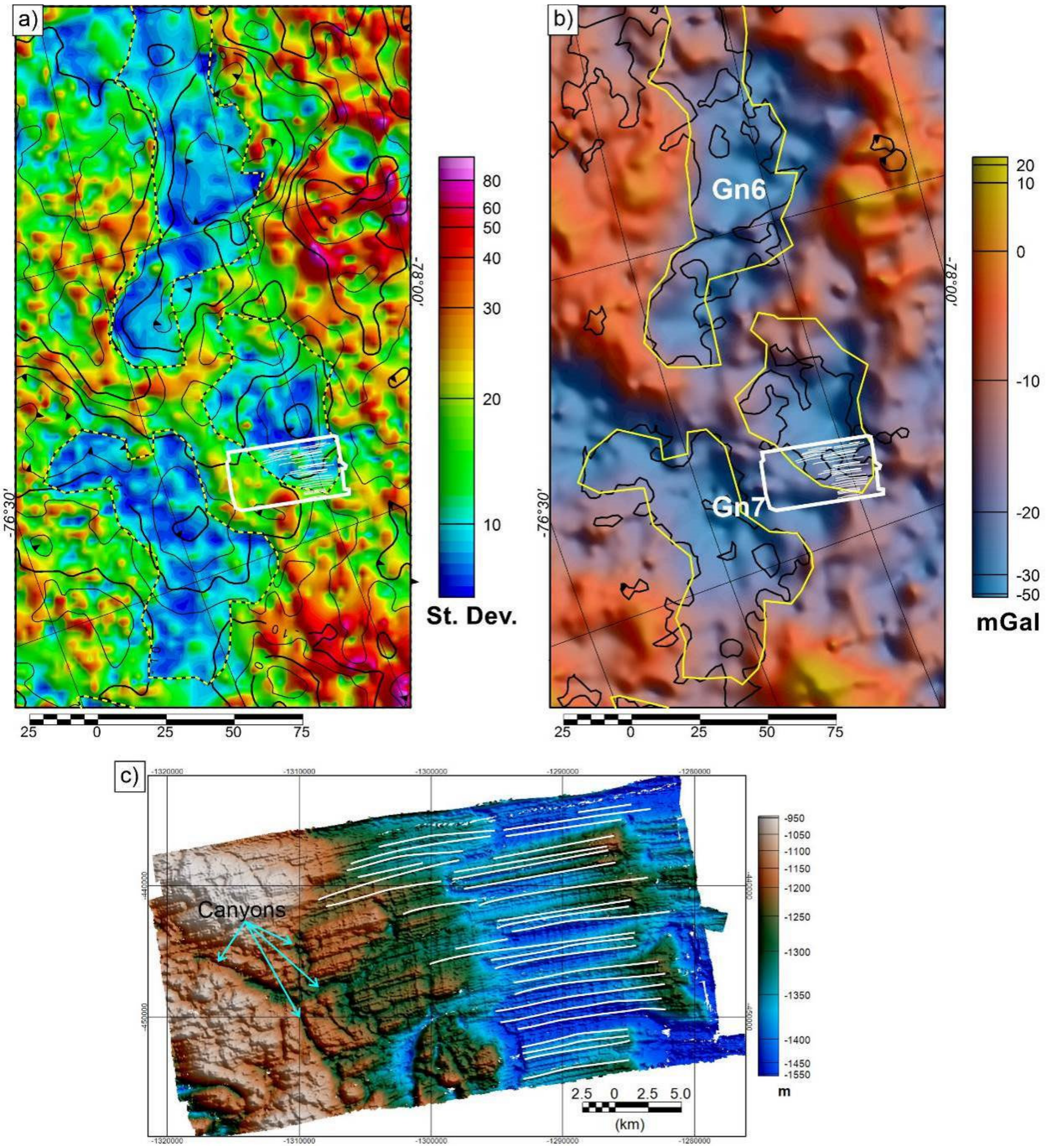

Fig. S8.

Example detail of bed roughness and Airy isostatic anomalies in area of inferred sedimentary basins (yellow outlines). a) Bed roughness map, based on along track standard deviation of bed elevation in 1 km moving window. Overlain contour shows Airy isostatic anomaly contoured at 5 mGal intervals. Note correspondence of negative gravity anomalies and regions of smooth bed. White box and lines locates high resolution radar image and Mega Scale Glacial Lineations (MSGL) formed of soft Till (*2*) shown in (c). b) Airy isostatic anomaly, with 10 m standard deviation contour, enclosing areas of low roughens as black line. c) Detailed swath radar image of subglacial topography (*31*). Note MSGL, highlighted in white, with amplitudes of up to 100 m, contrasting with shorter more localised lineations and sinuous canyons indicative of channelized water flow, over the adjacent highlands.

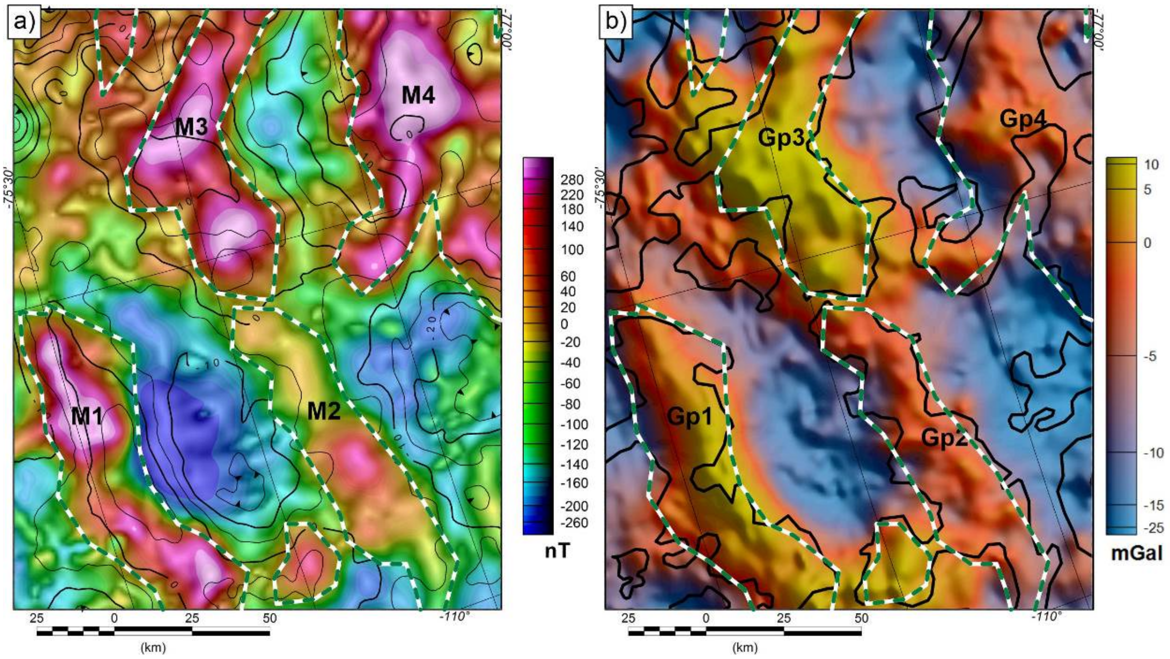

Fig. S9.

Example detail of magnetic and gravity anomalies over proposed mafic intrusions (green dashed lines). a) Aeromagnetic anomalies shaded with TDX enhancement of source edges (darker shading). Contours show Airy isostatic anomaly at 5 mGal interval. b) Airy isostatic anomaly with zero magnetic tilt angle (black line) definition of magnetic source edges. Note in this region positive gravity anomalies generally fall within magnetic tilt contour definition of source bodies.

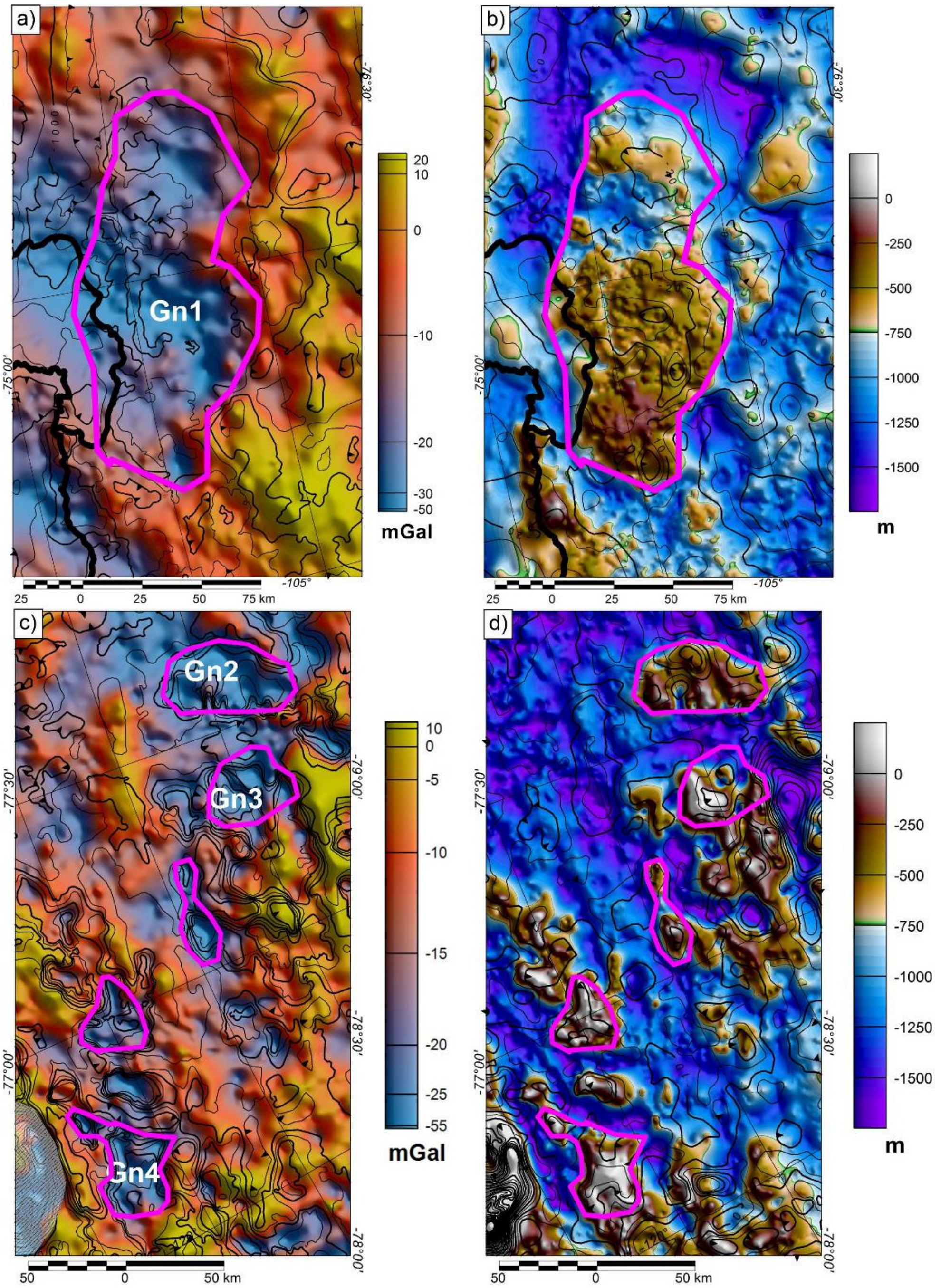

Fig. S10.

Detail over interpreted felsic granitoids (pink outlines). Only anomalies where positive topography and negative Airy isostatic anomalies coincide are inferred to be felsic granitoids. a) Negative Airy isostatic anomaly adjacent to Pine Island Glacier. Contours show bed elevation at 250 m interval. b) Subglacial topography, with 5 mGal contour of Airy isostatic anomaly. c) Inboard gravity anomalies. Contours show bed elevation at 250 m interval. d) Subglacial topography with 5 mGal contour of gravity anomalies.

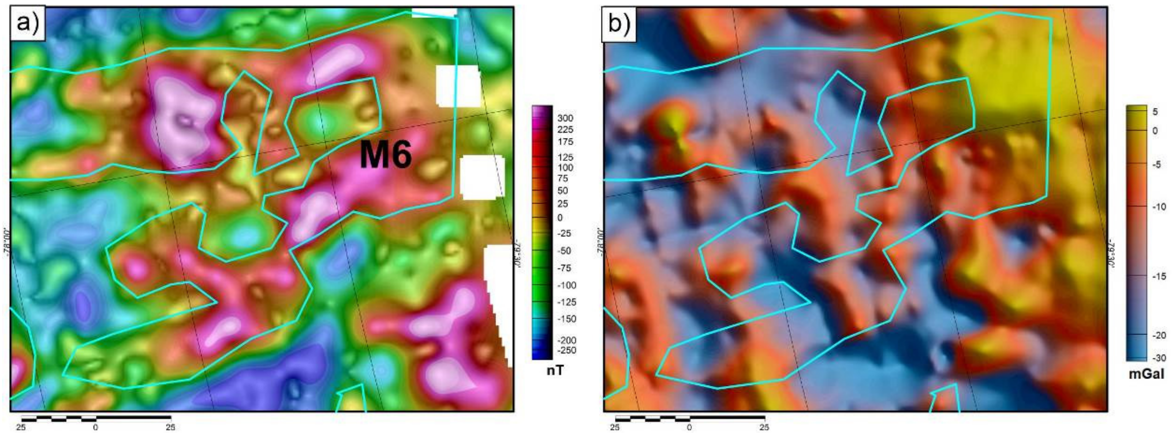

Fig. S11.

Detail of magnetic and gravity anomalies over proposed intermediate composition bodies (pale blue line). a) magnetic anomalies shaded with TDX enhancement of source edges (darker shading). b) Airy isostatic anomaly map. Note there is little correspondence between the magnetic and gravity anomalies.

Table S1.
Details of aerogeophysical surveys utilized in this paper.

**Survey & dates**	**Survey aircraft & design**	**Radar depth-sounding system**	**Gravity system**	**Magnetic system**
BBAS 2004/05	Twin Otter: Constant elevation blocks, 30 km spaced lines and tie-lines.	BAS PASIN depth sounder.	LaCoste & Romberg Air-Sea in a stabilised platform 9-km half-width filter.	Wing-tip Cs magnetometers.
AGASEA 2004/05	Twin Otter: Constant elevation blocks, 15 km spaced lines and tie-lines.	UTIG HICARS system.	LaCoste & Romberg Air-Sea in a stabilised platform 9-km half-width filter.	Towed magnetometer.
OIB 2009-2019	DC-8, P-3B: Draped ~450 m above surface. Detailed survey 2.5 km line spacing and regional lines.	CReSIS MCoRDS system.	Sander AIRGrav system. 5.2 km half-wavelength.	None
RV Polarstern expeditions ANT-XXIII/4 (2006) and ANT-XXVI/3 (2010)	BO-105 helicopters & ship-borne sensors. 100 m above sea surface with ~10 km line spacing.	None	None	Helicopter towed caesium-vapour magnetometer, and fluxgate on ship.
ITGC 2018/19 and 2019/20	Twin Otter: Draped ~400 m above ice surface.	CReSIS snow radar (2018/19) BAS PASIN2 depth sounder (2019/20).	iMAR iCORUS strapdown gravity system. ~5 km half-wavelength.	Wing-tip Cs magnetometers.

REFERENCES AND NOTES

1. T. A. Scambos, R. E. Bell, R. B. Alley, S. Anandakrishnan, D. H. Bromwich, K. Brunt, K. Christianson, T. Creyts, S. B. Das, R. DeConto, P. Dutrieux, H. A. Fricker, D. Holland, J. MacGregor, B. Medley, J. P. Nicolas, D. Pollard, M. R. Siegfried, A. M. Smith, E. J. Steig, L. D. Trusel, D. G. Vaughan, P. L. Yager, How much, how fast?: A science review and outlook for research on the instability of Antarctica's Thwaites Glacier in the 21st century. *Global Planet. Change* **153**, 16–34 (2017).

2. R. B. Alley, N. Holschuh, D. R. MacAyeal, B. R. Parizek, L. Zoet, K. Riverman, A. Muto, K. Christianson, E. Clyne, S. Anandakrishnan, N. Stevens; GHOST Collaboration, Bedforms of Thwaites Glacier, West Antarctica: Character and origin. *J. Geophys. Res. Earth Surf.* **126**, e2021JF006339 (2021).

3. P. Milillo, E. Rignot, P. Rizzoli, B. Scheuchl, J. Mouginot, J. Bueso-Bello, P. Prats-Iraola, Heterogeneous retreat and ice melt of Thwaites Glacier, West Antarctica. *Sci. Adv.* **5**, eaau3433 (2019).

4. R. E. Bell, D. D. Blankenship, C. A. Finn, D. L. Morse, T. A. Scambos, J. M. Brozena, S. M. Hodge, Influence of subglacial geology on the onset of a West Antarctic ice stream from aerogeophysical observations. *Nature* **394**, 58–62 (1998).

5. L. E. Peters, S. Anandakrishnan, R. B. Alley, J. P. Winberry, D. E. Voigt, A. M. Smith, D. L. Morse, Subglacial sediments as a control on the onset and location of two Siple Coast ice streams, West Antarctica. *J. Geophys. Res. Solid Earth* **111** (2006).

6. J. C. Behrendt, The aeromagnetic method as a tool to identify Cenozoic magmatism in the West Antarctic Rift System beneath the West Antarctic Ice Sheet—A review; Thiel subglacial volcano as possible source of the ash layer in the WAISCORE. *Tectonophysics* **585**, 124–136 (2013).

7. E. Quartini, D. D. Blankenship, D. A. Young, Chapter 7.5 Active subglacial volcanism in West Antarctica. *Geol. Soc. Lond. Mem.* **55**, 785–803 (2021).

8. M. van Wyk de Vries, R. G. Bingham, A. S. Hein, A new volcanic province: An inventory of subglacial volcanoes in West Antarctica. *Geol. Soc. Lond. Spec. Publ.* **461**, 231–248 (2018).

9. R. Dziadek, F. Ferraccioli, K. Gohl, High geothermal heat flow beneath Thwaites Glacier in West Antarctica inferred from aeromagnetic data. *Commun. Earth Environ.* **2**, 162 (2021).

10. P. Christoffersen, M. Bougamont, S. P. Carter, H. A. Fricker, S. Tulaczyk, Significant groundwater contribution to Antarctic ice streams hydrologic budget. *Geophys. Res. Lett.* **41**, 2003–2010 (2014).

11. M. J. Siegert, B. Kulessa, M. Bougamont, P. Christoffersen, K. Key, K. R. Andersen, A. D. Booth, A. M. Smith, Antarctic subglacial groundwater: A concept paper on its measurement and potential influence on ice flow. *Geol. Soc. Lond. Spec. Publ.* **461**, 197–213 (2018).

12. T. A. Jordan, T. R. Riley, C. S. Siddoway, The geological history and evolution of West Antarctica. *Nat. Rev. Earth Environ.* **1**, 117–133 (2020).

13. A. J. Tulloch, D. L. Kimbrough, S. E. Johnson, S. R. Paterson, J. M. Fletcher, G. H. Girty, D. L. Kimbrough, A. Martín-Barajas, Paired plutonic belts in convergent margins and the development of high Sr/Y magmatism: Peninsular Ranges Batholith of Baja California and Median Batholith of New Zealand, in *Tectonic Evolution of Northwestern Mexico and the Southwestern USA* (Geological Society of America, 2003), vol. 374.

14. R. J. Pankhurst, S. D. Weaver, J. D. Bradshaw, B. C. Storey, T. R. Ireland, Geochronology and geochemistry of pre-Jurassic superterranes in Marie Byrd Land, Antarctica. *J. Geophys. Res. Solid Earth* **103**, 2529–2547 (1998).

15. S. B. Mukasa, I. W. D. Dalziel, Marie Byrd Land,West Antarctica: Evolution of Gondwana's Pacific margin constrained by zircon U-Pb geochronology and feldspar common-Pb isotopic compositions. *Geol. Soc. Am. Bull.* **112**, 611–627 (2000).

16. A. J. Tulloch, N. Mortimer, T. R. Ireland, T. E. Waight, R. Maas, J. M. Palin, T. Sahoo, H. Seebeck, M. W. Sagar, A. Barrier, R. E. Turnbull, Reconnaissance basement geology and tectonics of South Zealandia. *Tectonics* **38**, 516–551 (2019).

17. T. R. Riley, M. J. Flowerdew, R. J. Pankhurst, P. T. Leat, I. L. Millar, C. M. Fanning, M. J. Whitehouse, A revised geochronology of Thurston Island, West Antarctica, and correlations along the proto-Pacific margin of Gondwana. *Antarct. Sci.* **29**, 47–60 (2017).

18. C. S. Siddoway, S. Richard, C. M. Fanning, B. P. Luyendyk, Origin and emplacement mechanisms for a middle Cretaceous gneiss dome, Fosdick Mountains, West Antarctica, in *Gneiss Domes in Orogeny,* D. L. Whitney, C. T. Teyssier, C. S. Siddoway, Eds. (Geological Society of America Special Paper 380, 2004), vol. 380, pp. 267–294.

19. F. Riefstahl, K. Gohl, B. Davy, K. Hoernle, N. Mortimer, C. Timm, R. Werner, K. Hochmuth, Cretaceous intracontinental rifting at the southern Chatham Rise margin and initialisation of seafloor spreading between Zealandia and Antarctica. *Tectonophysics* **776**, 228298 (2020).

20. K. Gohl, A. Denk, G. Eagles, F. Wobbe, Deciphering tectonic phases of the Amundsen Sea Embayment shelf, West Antarctica, from a magnetic anomaly grid. *Tectonophysics* **585**, 113–123 (2013).

21. F. Ferraccioli, E. Bozzo, D. Damaske, Aeromagnetic signatures over western Marie Byrd Land provide insight into magmatic arc basement, mafic magmatism and structure of the Eastern Ross Sea Rift flank. *Tectonophysics* **347**, 139–165 (2002).

22. R. Granot, J. Dyment, Late Cenozoic unification of East and West Antarctica. *Nat. Commun.* **9**, 3189 (2018).

23. T. A. Jordan, F. Ferraccioli, D. G. Vaughan, J. W. Holt, H. Corr, D. D. Blankenship, T. M. Diehl, Aerogravity evidence for major crustal thinning under the Pine Island Glacier region (West Antarctica). *Geol. Soc. Am. Bull.* **122**, 714–726 (2010).

24. W. LeMasurier, Shield volcanoes of Marie Byrd Land, West Antarctic rift: Oceanic island similarities, continental signature, and tectonic controls. *Bull. Volcanol.* **75**, 726 (2013).

25. E. M. Lucas, D. Soto, A. A. Nyblade, A. J. Lloyd, R. C. Aster, D. A. Wiens, J. P. O'Donnell, G. W. Stuart, T. J. Wilson, I. W. Dalziel, J. P. Winberry, A. D. Huerta, P- and S-wave

velocity structure of central West Antarctica: Implications for the tectonic evolution of the West Antarctic rift system. *Earth Planet. Sci. Lett.* **546**, 116437 (2020).

26. J. P. O'Donnell, G. W. Stuart, A. M. Brisbourne, K. Selway, Y. Yang, G. A. Nield, P. L. Whitehouse, A. A. Nyblade, D. A. Wiens, R. C. Aster, S. Anandakrishnan, A. D. Huerta, T. Wilson, J. P. Winberry, The uppermost mantle seismic velocity structure of West Antarctica from Rayleigh wave tomography: Insights into tectonic structure and geothermal heat flow. *Earth Planet. Sci. Lett.* **522**, 219–233 (2019).

27. T. M. Damiani, T. A. Jordan, F. Ferraccioli, D. A. Young, D. D. Blankenship, Variable crustal thickness beneath Thwaites Glacier revealed from airborne gravimetry, possible implications for geothermal heat flux in West Antarctica. *Earth Planet. Sci. Lett.* **407**, 109–122 (2014).

28. C. Ramirez, A. Nyblade, S. E. Hansen, D. A. Wiens, S. Anandakrishnan, R. C. Aster, A. D. Huerta, P. Shore, T. Wilson, Crustal and upper-mantle structure beneath ice-covered regions in Antarctica from S-wave receiver functions and implications for heat flow. *Geophys. J. Int.* **204**, 1636–1648 (2016).

29. T. M. Diehl, J. W. Holt, D. D. Blankenship, D. A. Young, T. A. Jordan, F. Ferraccioli, First airborne gravity results over the Thwaites Glacier catchment, West Antarctica. *Geochem. Geophys. Geosyst.* **9** (2008).

30. I. Joughin, S. Tulaczyk, J. L. Bamber, D. Blankenship, J. W. Holt, T. Scambos, D. G. Vaughan, Basal conditions for Pine Island and Thwaites Glaciers,West Antarctica, determined using satellite and airborne data. *J. Glaciology* **55**, 245–257 (2009).

31. N. Holschuh, K. Christianson, J. Paden, R. B. Alley, S. Anandakrishnan, Linking postglacial landscapes to glacier dynamics using swath radar at Thwaites Glacier, Antarctica. *Geology* **48**, 268–272 (2020).

32. E. R. Clyne, S. Anandakrishnan, A. Muto, R. B. Alley, D. E. Voigt, Interpretation of topography and bed properties beneath Thwaites Glacier, West Antarctica using seismic reflection methods. *Earth Planet. Sci. Lett.* **550**, 116543 (2020).

33. A. Muto, S. Anandakrishnan, R. B. Alley, H. J. Horgan, B. R. Parizek, S. Koellner, K. Christianson, N. Holschuh, Relating bed character and subglacial morphology using seismic data from Thwaites Glacier, West Antarctica. *Earth Planet. Sci. Lett.* **507**, 199–206 (2019).

34. H. N. Pollack, S. J. Hurter, J. R. Johnson, Heat flow from the Earth's interior: Analysis of the global data set. *Rev. Geophys.* **31**, 267–280 (1993).

35. D. M. Schroeder, D. D. Blankenship, D. A. Young, E. Quartini, Evidence for elevated and spatially variable geothermal flux beneath the West Antarctic Ice Sheet. *Proc. Natl. Acad. Sci. U.S.A.* **111**, 9070–9072 (2014).

36. W. Shen, D. A. Wiens, A. J. Lloyd, A. A. Nyblade, A geothermal heat flux map of Antarctica empirically constrained by seismic structure. *Geophys. Res. Lett.* **47**, e2020GL086955 (2020).

37. R. G. Bingham, M. J. Siegert, Radar-derived bed roughness characterization of Institute and Möller ice streams, West Antarctica, and comparison with Siple Coast ice streams. *Geophys. Res. Lett.* **34**, L21504 (2007).

38. R. J. Blakely, *Potential Theory in Gravity and Magnetic Applications* (Cambridge Univ. Press, 2009).

39. S. Anandakrishnan, J. P. Winberry, Antarctic subglacial sedimentary layer thickness from receiver function analysis. *Global Planet. Change* **42**, 167–176 (2004).

40. M. G. Laird, J. D. Bradshaw, The break-up of a long-term relationship: The Cretaceous separation of New Zealand from Gondwana. *Gondw. Res.* **7**, 273–286 (2004).

41. J. I. Raine, E. M. Kennedy, A. G. Griffin, R. Sykes, C. D. Clowes, Materials for improved assessment of the petroleum source potential of New Zealand coaly rocks, 1: Mid-Cretaceous stratigraphy, coal abundance, flora, and climate (GNS Science, 2018).

42. J. P. Klages, U. Salzmann, T. Bickert, C.-D. Hillenbrand, K. Gohl, G. Kuhn, S. M. Bohaty, J. Titschack, J. Müller, T. Frederichs, T. Bauersachs, W. Ehrmann, T. van de Flierdt, P. S. Pereira, R. D. Larter, G. Lohmann, I. Niezgodzki, G. Uenzelmann-Neben, M. Zundel, C. Spiegel, C. Mark, D. Chew, J. E. Francis, G. Nehrke, F. Schwarz, J. A. Smith, T. Freudenthal, O. Esper, H. Pälike, T. A. Ronge, R. Dziadek; Science Team of Expedition PS104, Temperate rainforests near the South Pole during peak Cretaceous warmth. *Nature* **580**, 81–86 (2020).

43. P. Simões Pereira, T. van de Flierdt, S. R. Hemming, T. Frederichs, S. J. Hammond, S. Brachfeld, C. Doherty, G. Kuhn, J. A. Smith, J. P. Klages, C.-D. Hillenbrand, The geochemical and mineralogical fingerprint of West Antarctica's weak underbelly: Pine Island and Thwaites glaciers. *Chem. Geol.* **550**, 119649 (2020).

44. E. L. Pierce, S. R. Hemming, T. Williams, T. van de Flierdt, S. N. Thomson, P. W. Reiners, G. E. Gehrels, S. A. Brachfeld, S. L. Goldstein, A comparison of detrital U–Pb zircon, ^{40}Ar/^{39}Ar hornblende, ^{40}Ar/^{39}Ar biotite ages in marine sediments off East Antarctica: Implications for the geology of subglacial terrains and provenance studies. *Earth Sci. Rev.* **138**, 156–178 (2014).

45. S. Rocchi, W. E. LeMasurier, G. Di Vincenzo, Oligocene to Holocene erosion and glacial history in Marie Byrd Land, West Antarctica, inferred from exhumation of the Dorrel Rock intrusive complex and from volcano morphologies. *Geol. Soc. Am. Bull.* **118**, 991–1005 (2006).

46. T. A. Jordan, F. Ferraccioli, N. Ross, H. F. J. Corr, P. T. Leat, R. G. Bingham, D. M. Rippin, A. le Brocq, M. J. Siegert, Inland extent of the Weddell Sea Rift imaged by new aerogeophysical data. *Tectonophysics* **585**, 137–160 (2013).

47. J. Lindow, P. J. J. Kamp, S. B. Mukasa, M. Kleber, F. Lisker, K. Gohl, G. Kuhn, C. Spiegel, Exhumation history along the eastern Amundsen Sea coast, West Antarctica, revealed by low-temperature thermochronology. *Tectonics* **35**, 2239–2257 (2016).

48. A. Kipf, N. Mortimer, R. Werner, K. Gohl, P. Van Den Bogaard, F. Hauff, K. Hoernle, Granitoids and dykes of the Pine Island Bay region, West Antarctica. *Antarct. Sci.* **24**, 473–484 (2012).

49. A. J. Tulloch, J. Ramezani, D. L. Kimbrough, K. Faure, A. H. Allibone, U-Pb geochronology of mid-Paleozoic plutonism in western New Zealand: Implications for S-type granite generation and growth of the east Gondwana margin. *Geol. Soc. Am. Bull.* **121**, 1236–1261 (2009).

50. N. Ross, R. G. Bingham, H. F. J. Corr, F. Ferraccioli, T. A. Jordan, A. Le Brocq, D. M. Rippin, D. Young, D. D. Blankenship, M. J. Siegert, Steep reverse bed slope at the grounding line of the Weddell Sea sector in West Antarctica. *Nat. Geosci.* **5**, 393–396 (2012).

51. F. S. McCormack, R. C. Warner, H. Seroussi, C. F. Dow, J. L. Roberts, A. Treverrow, Modeling the deformation regime of Thwaites Glacier, West Antarctica, using a simple flow relation for ice anisotropy (ESTAR). *J. Geophys. Res. Earth Surf.* **127**, e2021JF006332 (2022).

52. G. T. Jarvis, D. P. McKenzie, Sedimentary basin formation with finite extension rates. *Earth Planet. Sci. Lett.* **48**, 42–52 (1980).

53. D. W. Waples, A new model for heat flow in extensional basins: Radiogenic heat, asthenospheric heat, and the McKenzie model. *Nat. Resour. Res.* **10**, 227–238 (2001).

54. P. T. Leat, T. A. Jordan, M. J. Flowerdew, T. R. Riley, F. Ferraccioli, M. J. Whitehouse, Jurassic high heat production granites associated with the Weddell Sea rift system, Antarctica. *Tectonophysics* **722**, 249–264 (2018).

55. C. D. Gustafson, K. Key, M. R. Siegfried, J. P. Winberry, H. A. Fricker, R. A. Venturelli, A. B. Michaud, A dynamic saline groundwater system mapped beneath an Antarctic ice stream. *Science* **376**, 640–644 (2022).

56. J. W. Holt, D. D. Blankenship, D. L. Morse, D. A. Young, M. E. Peters, S. D. Kempf, T. G. Richter, A. P. M. Vaughan, H. Corr, New boundary conditions for the West Antarctic Ice Sheet: Subglacial topography of the Thwaites and Smith glacier catchments. *Geophys. Res. Lett.* **33** (2006).

57. D. G. Vaughan, H. F. J. Corr, F. Ferraccioli, N. Frearson, A. O'Hare, D. Mach, J. W. Holt, D. D. Blankenship, D. L. Morse, D. A. Young, New boundary conditions for the West Antarctic ice sheet: Subglacial topography beneath Pine Island Glacier. *Geophys. Res. Lett.* **33** (2006).

58. M. Morlighem, E. Rignot, T. Binder, D. Blankenship, R. Drews, G. Eagles, O. Eisen, F. Ferraccioli, R. Forsberg, P. Fretwell, V. Goel, J. S. Greenbaum, H. Gudmundsson, J. Guo, V. Helm, C. Hofstede, I. Howat, A. Humbert, W. Jokat, N. B. Karlsson, W. S. Lee, K. Matsuoka, R. Millan, J. Mouginot, J. Paden, F. Pattyn, J. Roberts, S. Rosier, A. Ruppel, H. Seroussi, E. C. Smith, D. Steinhage, B. Sun, M. R. V. D. Broeke, T. D. V. Ommen, M. V. Wessem, D. A. Young, Deep glacial troughs and stabilizing ridges unveiled beneath the margins of the Antarctic ice sheet. *Nat. Geosci.* **13**, 132–137 (2020).

59. T. A. Jordan, D. Porter, K. Tinto, R. Millan, A. Muto, K. Hogan, R. D. Larter, A. G. C. Graham, J. D. Paden, New gravity-derived bathymetry for the Thwaites, Crosson, and Dotson ice shelves revealing two ice shelf populations. *Cryosphere* **14**, 2869–2882 (2020).

60. D. M. Rippin, R. G. Bingham, T. A. Jordan, A. P. Wright, N. Ross, H. F. J. Corr, F. Ferraccioli, A. M. Le Brocq, K. C. Rose, M. J. Siegert, Basal roughness of the Institute and Möller Ice Streams,West Antarctica: Process determination and landscape interpretation. *Geomorphology* **214**, 139–147 (2014).

61. A. V. Golynsky, F. Ferraccioli, J. K. Hong, D. A. Golynsky, R. R. B. Frese, D. A. Young, D. D. Blankenship, J. W. Holt, S. V. Ivanov, A. V. Kiselev, V. N. Masolov, G. Eagles, K. Gohl, W. Jokat, D. Damaske, C. Finn, A. Aitken, R. E. Bell, E. Armadillo, T. A. Jordan, J. S.

Greenbaum, E. Bozzo, G. Caneva, R. Forsberg, M. Ghidella, J. Galindo-Zaldivar, F. Bohoyo, Y. M. Martos, Y. Nogi, E. Quartini, H. R. Kim, J. L. Roberts, New magnetic anomaly map of the Antarctic. *Geophys. Res. Lett.* **45**, 6437–6449 (2018).

62. V. Baranov, H. Naudy, Numerical calculation of the formula of reduction to the magnetic pole. *Geophysics* **29**, 67–79 (1964).

63. G. R. J. Cooper, D. R. Cowan, Enhancing potential field data using filters based on the local phase. *Comput. Geosci.* **32**, 1585–1591 (2006).

64. A. Salem, S. Williams, J. D. Fairhead, D. Ravat, R. Smith, Tilt-depth method: A simple depth estimation method using first-order magnetic derivatives. *Lead. Edge*, **26**, 1489–1624 (2007).

65. I. G. Roy, Tilt angle interpretation of dipping fault model. *J. Appl. Geophys.* **98**, 33–43 (2013).

66. J. D. Philips. (Society of Exploration Geophysicists, 2002 Technical Program Expanded Abstracts, 2002), p. 4.

67. A. B. Reid, J. M. Allsop, H. Granser, A. J. Millet, I. W. Somerton, Magnetic interpretation in three dimensions using Euler deconvolution. *Geophysics* **55**, 10–131 (1990).

68. R. G. Elllis, B. de Wet, I. N. Macleod, Inversion of magnetic data from remanent and induced sources. *ASEG Extend. Abstr.* **2012**, 1–4 (2012).

69. E. Rignot, J. Mouginot, B. Scheuchl, MEaSUREs InSAR-Based Antarctica Ice Velocity Map, Version 2 (NASA National Snow and Ice Data Center Distributed Active Archive Center, 2017); http://dx.doi.org/10.5067/D7GK8F5J8M8R.

70. B. E. Smith, N. Gourmelen, A. Huth, I. Joughin, Connected subglacial lake drainage beneath Thwaites Glacier, West Antarctica. *Cryosphere* **11**, 451–467 (2017).

71. J. Paden, J. Li, C. Leuschen, F. Rodriguez-Morales, R. Hale, IceBridge MCoRDS L2 Ice Thickness, Version 1 (NASA National Snow and Ice Data Center Distributed Active Archive Center, 2010, updated 2021); https://doi.org/10.5067/GDQ0CUCVTE2Q.

72. K. Tinto, R. Bell, J. R. Cochran, IceBridge Sander AIRGrav L1B Geolocated Free Air Gravity Anomalies, Version 1 (NASA National Snow and Ice Data Center Distributed Active Archive Center, 2010, updated 2019); https://doi.org/10.5067/R1RQ6NRIJV89.

73. T. Jordan, C. Robinson, D. Porter, Processed line aeromagnetic data over the Thwaites Glacier region (2018/19 season) (Natural Environment Research Council, UK Research & Innovation, UK Polar Data Centre, 2020); https://doi.org/10.5285/776612D1-573C-49C4-AFF5-23B0FBA48271.

74. T. A. Jordan, C. Robinson, Processed line aeromagnetic data over the Thwaites Glacier region (2019/2020 season) (version 1.0) (NERC EDS UK Polar Data Centre, 2021); https://doi.org/10.5285/EB6BF8DF-EA87-4C3C-8F27-33FA8D5AC663.

75. T. A. Jordan, C. Robinson, Processed line aerogravity data over the Thwaites Glacier region (2019/20 season) (version 1.0) (NERC EDS, UK Polar Data Centre, 2021); https://doi.org/10.5285/AF14076F-F0C4-479F-B38F-6AA0B7C7D314.

76. T. A. Jordan, C. Robinson, D. Porter, C. Locke, K. Tinto, Processed line aerogravity data over the Thwaites Glacier region (2018/19 season) (Natural Environment Research Council, UK Research & Innovation, UK Polar Data Centre, 2020); https://doi.org/10.5285/B9B28A35-8620-4182-BF9C-638800B6679B.

77. H. G. Miller, V. Singh, Potential field tilt—A new concept for location of potential field sources. *J. Appl. Geophys.* **32**, 213–217 (1994).

78. T. A. Jordan, D. Becker, Investigating the distribution of magmatism at the onset of Gondwana breakup with novel strapdown gravity and aeromagnetic data. *Phys. Earth Planet. Inter.* **282**, 77–88 (2018).

79. P. Fretwell, H. D. Pritchard, D. G. Vaughan, J. Bamber, N. Barrand, R. Bell, C. Bianchi, R. Bingham, D. Blankenship, G. Casassa, D. Callens, H. Conway, A. J. Cook, H. F. J. Corr, D. Damaske, V. Damm, F. Ferraccioli, R. Forsberg, S. Fujita, P. Gogineni, J. A. Griggs, R. Hindmarsh, P. Holmlund, J. W. Holt, R. W. Jacobel, A. Jenkins, W. Jokat, T. A. Jordan, E. C. King, J. Kohler, W. Krabill, R. Riger-Kusk, K. A. Langley, G. Leitchenkov, C. Leuschen, B. P. Luyendyk, K. Matsuoka, Y. Nogi, O. A. Nost, S. Popov, E. Rignot, D. M. Rippin, A. Riviera, J. Roberts, N. Ross, M. J. Siegert, A. M. Smith, D. Steinhage, M. Studinger, B. Sun, B. K. Tinto, B. C. Welch, D. A. Young, C. Xiangbin, A. Zirizzotti, Bedmap2: Improved ice bed, surface and thickness datasets for Antarctica. *Cryosphere* **7**, 375–393 (2013).

80. R. R. B. von Frese, W. J. Hinze, L. W. Braile, A. J. Luca, Spherical-Earth gravity and magnetic anomaly modeling by Gauss-Legendre quadrature integration. *J. Geophys.* **49**, 234–242 (1981).

81. C. K. Dunham, J. P. O'Donnell, G. W. Stuart, A. M. Brisbourne, S. Rost, T. A. Jordan, A. A. Nyblade, D. A. Wiens, R. C. Aster, A joint inversion of receiver function and Rayleigh wave phase velocity dispersion data to estimate crustal structure in West Antarctica. *Geophys. J. Int.* **223**, 1644–1657 (2020).

82. M. S. Zhdanov, *Geophysical Inverse Theory and Regularization Problems* (Elsevier Science Publishing Co. Inc., 2002).
